# A Mathematical Perspective on the Influence of Allee Effects in Oncolytic Virotherapy

**DOI:** 10.3390/math13050744

**Published:** 2025-02-25

**Authors:** Eymard Hernández-López, Jin Wang

**Affiliations:** 1 Department of Mathematics, University of Tennessee at Chattanooga, Chattanooga, TN 37403, USA; 2 Postgraduate and Research, TecNM-TESOEM, Estado de México 56400, Mexico

**Keywords:** dynamical systems, tumor eradication, virotherapy, bifurcation diagrams, Hopf bifurcation, logistic growth, allee effects, 92-10, 34D20, 34C23

## Abstract

This article is concerned with the mathematical modeling of cancer virotherapy, emphasizing the impact of Allee effects on tumor cell growth. We propose a modeling framework that describes the complex interaction between tumor cells and oncolytic viruses. The efficacy of this therapy against cancer is mathematically investigated. The analysis involves linear and logistic growth scenarios coupled with different Allee effects, including weak, strong, and hyper Allee forms. Critical points are identified, and their existence and stability are analyzed using dynamical system theories and bifurcation techniques. Also, bifurcation diagrams and numerical simulations are utilized to verify and extend analytical results. It is observed that Allee effects significantly influence the stability of the system and the conditions necessary for tumor control and eradication.

## 1. Introduction

Virotherapy, an emerging strategy in cancer treatment, uses oncolytic viruses to infect and destroy tumor cells while sparing healthy tissues. With the ability to cause tumor destruction through cancer-selective viruses based on various genetic mechanisms and delivery techniques, this therapeutic approach has gained attention as a promising complement or alternative to conventional treatment strategies such as chemotherapy and radiation therapy. However, there are several challenges currently associated with virotherapy that may limit its application in cancer treatment [1–4]. In particular, the interaction between oncolytic viruses and cancer cells within the tumor microenvironment is not well understood at present.

Tumor cells exhibit complex growth patterns inside the human body that may involve linear and nonlinear dynamics. Meanwhile, the Allee effect, a phenomenon observed initially in ecology that describes how populations of macroorganisms [5–8] or microorganisms [9,10] exhibit reduced growth rates at low densities, also potentially plays a crucial role in cancer development [11–13]. Like populations in ecosystems, the Allee effect in tumor cells suggests that cell cooperation is key to their growth and cancer proliferation at low densities. Understanding this mechanism would help in proposing new approaches to disrupt cell communication and cooperation by preventing the critical population from growing efficiently. In this sense, tumor cells have limited growth due to a lack of sufficient autocrine signals, as there is evidence of dependence on the concentration of autocrine growth factors produced by themselves [14–17]. Growth factors may be platelet-derived growth factor PDGF. Normal cells need to receive signals from their environment to grow; however, tumor cells may produce both ligands and receptors, thus creating an autocrine loop [18].

Several studies have noted that the Allee effect may also influence cancer recurrence after treatment, pointing out that they can proliferate even at this stage, reaching their critical density more quickly. This may be due to the ability of tumor cells to manipulate their environment through autocrine cycling or as in the process of secretion of tumor TAF Angiogenesis Factor, which eventually activates endothelial cells to obtain nutrients through mini-vascularizations; this angiogenesis phenomenon could increase the capacity of tumor growth rates [19]. Given that normal cells have a different growth rate compared to cancer cells [20,21], understanding the cooperative behavior of cancer cells, similar to the Allee effect in tumor recurrence, could contribute to the design of therapies, in this case by modulating endothelial cell migration, anti-angiogenesis treatment in tumors, or anti-autocrine treatments [22–25].

Such complex tumor cell growth dynamics, coupled with the spread and replication of oncolytic viruses and the host–virus interaction, will directly impact the outcome of virotherapy. Mathematical modeling offers a powerful theoretical tool to understand the rich dynamics involved in tumor virotherapy, allowing a meaningful prediction of the efficacy of treatment and the optimization of key parameters [2,4,26–32]. Cancer modeling and simulation have advanced rapidly over the last few decades, showing a strong potential to quantify tumor dynamics and guide therapeutic development in oncology, including in diagnosis, treatment, and tumor management. In particular, the development of mathematical models allows the exploration of complex cellular–viral dynamics and the evaluation of fundamental strategies to control tumor growth through viral infection [31,33–37]. This research area may integrate mathematical biology with promising clinical applications, utilizing concepts and modeling techniques from other fields such as epidemiology (for viral infection) and ecology (for population growth and Allee effects) [7,38,39]. Recent modeling studies, for example, have combined differential equations and stochastic rules to capture the interactions between tumor cells, oncolytic viruses, and immune responses [40,41]. Furthermore, incorporating Allee effects into oncology models could demonstrate how population thresholds affect tumor stability and dynamic transitions and allow the exploration of different scenarios in virotherapy towards tumor eradication.

The present paper proposes a theoretical framework based on ordinary differential equations that incorporates multiple types of Allee effects (weak, strong, and hyper) and different patterns of tumor cell growth dynamics (linear and logistic). We plan to investigate the various scenarios involved in tumor–virus interactions thoroughly. The models representing these different scenarios are analyzed from the perspective of bifurcations, allowing the identification of critical conditions for the stability of equilibrium points and the occurrence of periodic oscillations. This study could provide quantitative tools to guide therapeutic design in controlling tumor growth by viruses.

This paper is organized as follows. Section 2 presents the general modeling framework. Sections 3 and 4 analyze linear and logistic growth patterns, respectively, each coupled with multiple types of Allee effects. Section 5 is devoted to bifurcation diagrams and numerical simulation results, followed by a discussion in Section 6. Finally, conclusions are drawn in Section 7.

## 2. Base Model

We propose a modeling framework that describes the interaction between normal tumor cells (denoted by T), infected tumor cells (denoted by I), and virions (denoted by V). These variables capture the key dynamics of the system, which aims to model the use of oncolytic viruses as an antitumor therapy with the goal of promoting tumor eradication in the patient.

In the following, we present a set of differential equations that describe these interactions between tumor cells and viruses, while incorporating fundamental parameters related to cancer dynamics and therapeutic effects of the viruses.

(1) $$T^{\prime }=\lambda g(T)A(T,\alpha ,\omega )-\beta TV,\;I^{\prime }=\beta TV-\mu I,\;V^{\prime }=pI-\delta V,$$

where the parameter λ is the rate at which new tumor cells are generated, β is the cell-virus contact rate, μ is the lysis rate of infected tumor cells, p is the viral replication rate, and δ is the viral removal rate. The function g(T) represents the growth of the tumor cell population, which will include linear and logistic growth patterns in our study. Meanwhile, the function A(T,α,ω) represents the Allee effects, where we will discuss the weak, strong, and hyper Allee effects separately. The initial conditions of the system (1) are T(0)≥0,I(0)≥0, and V(0)≥0.

**Figure 1.**
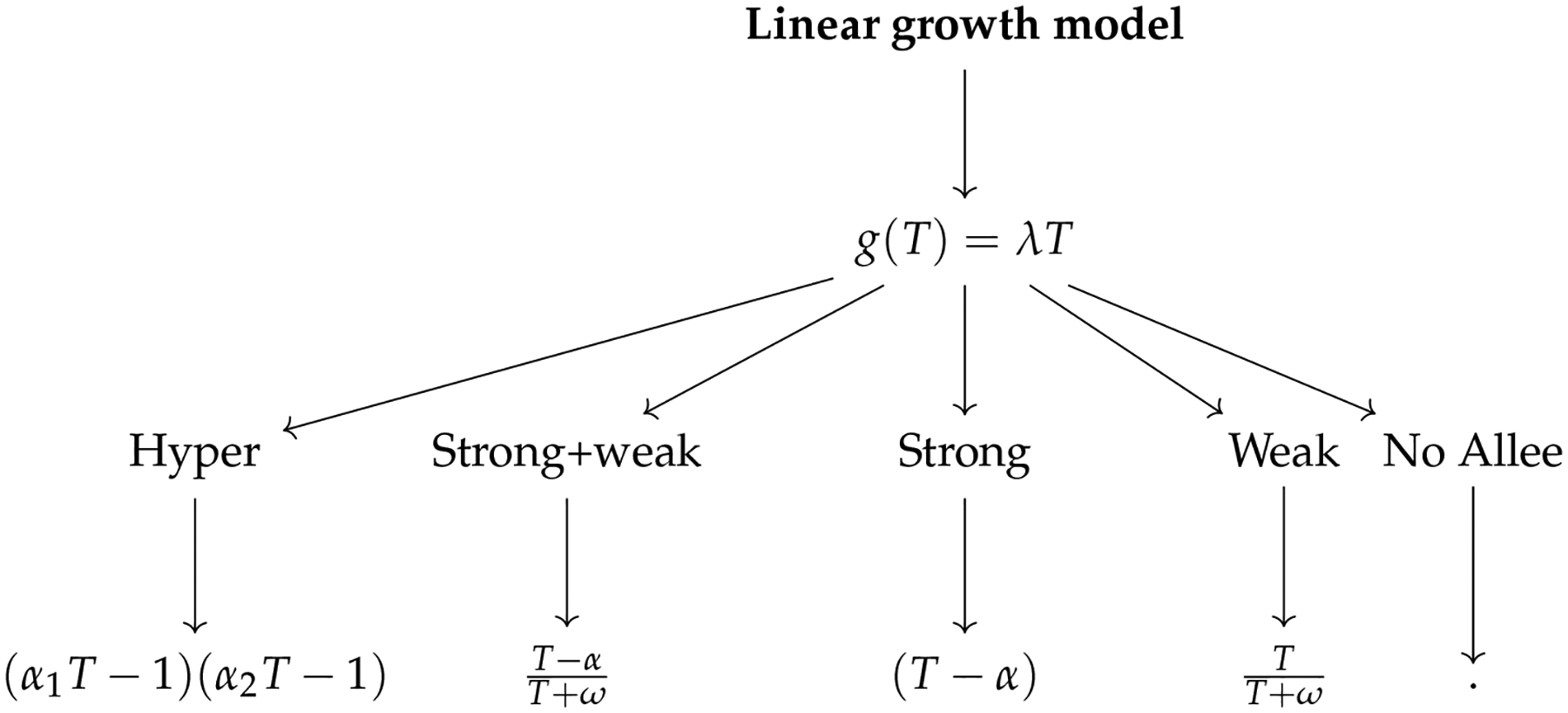

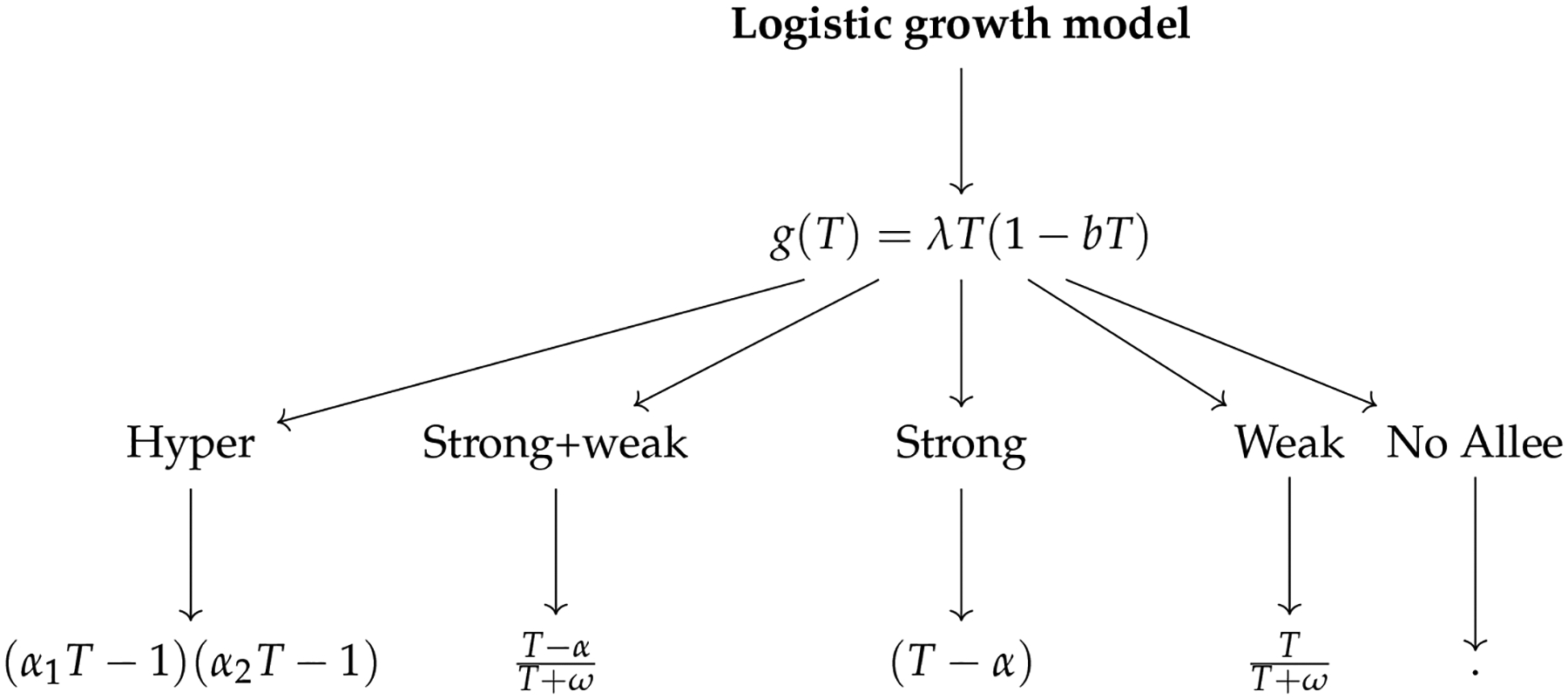
Functional forms of the Allee effect, with references to the corresponding bifurcation diagrams.

In the analysis presented below, we will discuss different forms of the functions g(T) and A(T,α,ω) that will represent the coupling of various growth dynamics and Allee effects. Specifically, we will analyze the linear growth model in Section 3 and the logistic growth model in Section 4. We will pay more attention to the logistic growth dynamics, which may be more realistic than the linear growth in that a carrying capacity for the tumor cells is incorporated. The analytic work will provide a foundation for the comparison in Section 5 concerning bifurcation diagrams and numerical continuation results. Figure 1 summarizes the functional forms of the growth dynamics and Allee effects analyzed in this work, as well as their connections to the bifurcation diagrams in Section 5. Furthermore, we emphasize that the Allee effects discussed throughout this paper will take various types and will be interpreted in a broad sense [11].

## 3. Linear Growth Model

### 3.1. Linear Growth with Weak and Strong Allee Effects

We examine a scenario that includes both Allee effects, as the sole consideration of the strong Allee effect produces dynamics similar to those observed when both effects are included simultaneously. This similarity is clearly illustrated in the bifurcation diagrams presented in Section 5.

So, we will consider the following system.

(2) $$T^{\prime }=\lambda TA(T,\alpha ,\omega )-\beta TV,\;I^{\prime }=\beta TV-\mu I,\;V^{\prime }=pI-\delta V,$$

where the term A(T,α,ω)=(T-α)/(T+ω) represents the weak and strong Allee effects simultaneously. Note that the case with weak Allee effect recovers when α=0. If we calculate the critical points, we obtain the trivial one (0, 0, 0), the free disease critical point for the strong Allee effect $\alpha ,P_{l\alpha }=(\alpha ,0,0)$, and the endemic point given by

$$P_{le_{2}}=\left(\frac{\delta \mu }{p\beta },\frac{\delta^{2}\lambda (\delta \mu -p\alpha \beta )}{p\beta (\delta \mu +p\beta \omega )},\frac{\lambda (\delta \mu -p\alpha \beta )}{p\beta (\delta \mu +p\beta \omega )}\right).$$

To evaluate the ability of a disease to spread within a population, it is essential to determine the basic reproductive number, $R_{\alpha }$. This parameter represents the average number of secondary cases an infection will generate during its infectious stage in a fully susceptible population. In models that incorporate the Allee effect, especially when a strong Allee effect is considered, the disease dynamics may present different behaviors compared to traditional models without this effect and even with a weak Allee effect.

By calculating the critical points of the system with the weak and strong Allee effect simultaneously, we have identified the critical point (α,0,0), where α denotes the level of the strong Allee effect. This critical point is particularly interesting as it represents an equilibrium in the system where population dynamics and disease spread interact significantly [42]. This point suggests specific conditions under which the tumor cell population infected by the virus can stabilize or extinguish.

Next, we will calculate the term $R_{\alpha }$ for this model, considering the level of the Allee effect defined by α. This calculation will allow us to analyze the stability of the critical point and better understand how the Allee effect influences the virus’s ability to be sustained in the tumor cell population. Using the next-generation matrix method [43], we obtain

$$F=\left(\begin{array}{cc} 0 & \alpha \beta \\ p & 0 \end{array}\right),\;V=\left(\begin{array}{cc} \mu & 0\\ 0 & \delta \end{array}\right)$$

and the basic reproductive number of the virus for system (2) for the strong Allee effect is given by

(3) $$R_{\alpha }=\rho \left(FV^{-1}\right)=\sqrt{\frac{p\alpha \beta }{\delta \mu }},$$

or

$$R_{\alpha }^{2}=\frac{p\alpha \beta }{\delta \mu }.$$

Note: In the base system (1), the basic reproductive number $R_{\alpha }$ corresponds to $R_{T*}=\frac{\beta pT^{*}}{\mu \delta }$, depending on the first component of the critical point $T^{*}$. Suppose that the basic virus reproduction number $R_{T^{*}}$ is less than 1 at the beginning of the infection. In that case, each virus-infected cancer cell produces, on average, fewer than one new infected cancer cell. Therefore, viral infection cannot spread within the cancer cell population, and the tumor returns to an uninfected state, leading to tumor persistence and eventual proliferation. However, if the basic reproduction number $R_{T^{*}}$ is greater than 1, at the beginning of the infection, each cancer cell infected by the virus produces, on average, more than one new infected cancer cell. The basic reproduction number is directly proportional to the level of the Allee effect, indicating that a higher Allee effect leads to a higher reproduction number, and consequently a more significant number of infected cancer cells, which would reduce the overall cancer cell population.

If we use this number at the endemic critical point $P_{le_{2}}$ we find that

(4) $$P_{le_{2}}=\left(\frac{\alpha }{R_{\alpha }^{2}},-\frac{\left(R_{\alpha }^{2}-1\right)\alpha^{2}\lambda }{R_{\alpha }^{4}\mu \left(\alpha +R_{\alpha }^{2}\omega \right)},-\frac{\left(R_{\alpha }^{2}-1\right)\alpha^{3}\lambda }{R_{\alpha }^{2}\beta \left(\alpha +R_{\alpha }^{2}\omega \right)}\right),$$

where the condition of existence is $0<R_{\alpha }^{2}<1$.

Once we have incorporated the strong and weak Allee effects in the system and determined the basic reproduction number $R_{\alpha }$ based on the disease-free critical point using the parameter α associated with the strong Allee effect, we proceed to analyze the behavior at the endemic point. In particular, we will explore a stability change by analyzing the Hopf bifurcation. The strong Allee effect α implies the existence of a minimum population threshold necessary to avoid extinction, while the weak Allee effect ω suggests that individual growth rate increases with population density without necessarily requiring a critical threshold.

In this analysis, we illustrate the conditions under which a Hopf bifurcation occurs in such a base system, with different Allee effects and, in this case, the linear population growth term. Subsequently, we will consider the logistic growth case. Also, this analysis will allow a better understanding of the transitions between steady state and oscillatory dynamics, providing a more complete picture of how Allee’s effects affect the stability and variability of the cancer cell populations in the system. By identifying these critical points, we seek to establish the key parameters that facilitate the emergence of sustained population cycles. This is fundamental for designing therapeutic strategies for considering virus-mediated tumor cell infection as an alternative to conventional treatments, which are also affected by Allee effects.

**Theorem 1**. *Consider the system given by* (2). *The following set*

(5) $$H=\left\{\left(\lambda ,\varsigma_{l_{WS_{1}}},\varsigma_{l_{WS_{2}}},R_{\alpha }^{2}\right)|\varsigma_{l_{WS_{1}}}\lambda -\varsigma_{l_{WS_{2}}}^{2}=0\right\},$$

*contains the symmetric-saddles and Hopf bifurcations in the point* $P_{{le}_{2}}$ (4), *where*

(6) $$\varsigma_{l_{WS_{1}}}=\alpha (\delta +\mu ){\left(\alpha^{2}+R_{\alpha }^{6}\omega -(\alpha +2)R_{\alpha }^{4}\omega +\alpha R_{\alpha }^{2}(-\alpha +\omega -1)\right)}^{2}\;\varsigma_{l_{WS_{2}}}=R_{\alpha }^{2}{\left(\alpha +R_{\alpha }^{2}\omega \right)}^{2}\left(-\alpha^{2}\left(\delta^{2}+\delta \mu +\mu^{2}\right)+R_{\alpha }^{6}\omega \left(-(\delta +\mu {)}^{2}\right)\right.\;+R_{\alpha }^{4}\omega \left((\alpha +2)\delta^{2}+(\alpha +4)\delta \mu +(\alpha +2)\mu^{2}\right)+\alpha R_{\alpha }^{2}\left(\delta^{2}(\alpha -\omega +1)\right.\;\left.\left.+\delta \mu (\alpha -\omega +2)+\mu^{2}(\alpha -\omega +1)\right)\right)$$

**Proof**. Taking the endemic point $P_{le_{2}}$ (4) and evaluating the Jacobian matrix A, in terms of parameters, we can then calculate its characteristic polynomial similar to Equation (7). Associated with A, this is

(7) $$p(\psi )=a_{0}\psi^{3}+a_{1}\psi^{2}+a_{2}\psi +a_{3},$$

in terms of the trace, the sum of all second-order diagonal minors of A are denoted by SimA, and the determinant of the matrix A, where $a_{0}=1,a_{1}=-\text{TrA},a_{2}=\text{Sim}A$, and $a_{3}=-\text{DetA}$. Then, we calculate the parameters $a_{1},a_{2}$, and $a_{3}$. We look for conditions where the $\left(a_{1}a_{2}-a_{3}\right)=0$ and we obtain the following set of parameters (5). □

Next, we calculate the stability region for the endemic point $P_{le_{2}}$ (4) in the model (2).

**Proposition 1**. *Let* $0<R_{\alpha }^{2}<1$ *and* $\varsigma_{l_{WS_{1}}}\lambda <\varsigma_{l_{WS_{2}}}^{2}$, *and then the endemic critical point* $P_{l_{e_{2}}}$ (4) *of a system* (2) *is locally asymptotically stable*.

**Proof**. According to the Hurwitz stability [44], $P_{le_{2}}$ (4) should be local asymptotically stable if $a_{1},a_{2},a_{3}>0$ and $a_{1}a_{2}-a_{3}>0$. First, we calculate the Jacobian matrix of the system evaluated at $P_{le_{2}}$ (4). From this matrix, we derive the characteristic polynomial and obtain the coefficients of the Hurwitz matrix, denoted as $a_{1},a_{2}$, and $a_{3}$. In this analysis, we consider the symmetric-saddle and Hopf bifurcations, incorporating a parameter ϵ, such that

(8) $$\lambda ≔\epsilon \frac{\varsigma_{l_{WS_{2}}}^{2}}{\varsigma_{l_{WS_{1}}}},$$

if we evaluate in ($a_{1}a_{2}-a_{3}$), then we find

(9) $$\left(a_{1}a_{2}-a_{3}\right)=\epsilon (\epsilon -1)\frac{\varsigma_{l_{WS_{2}}}^{2}}{\varsigma_{l_{WS_{1}}}}.$$

If ϵ=1, the we have the Hopf bifurcation, but if ϵ>1 then $\left(a_{1}a_{2}-a_{3}\right)>0$ for $a_{1},a_{2},a_{3}>0$. For all, $P_{le_{2}}$ (4) is locally asymptotically stable under $0<R_{\alpha }^{2}<1$ and $\varsigma_{l_{WS_{1}}}\lambda <\epsilon \varsigma_{l_{WS_{2}}}^{2}$, with ϵ>1. □

**Figure 2.**
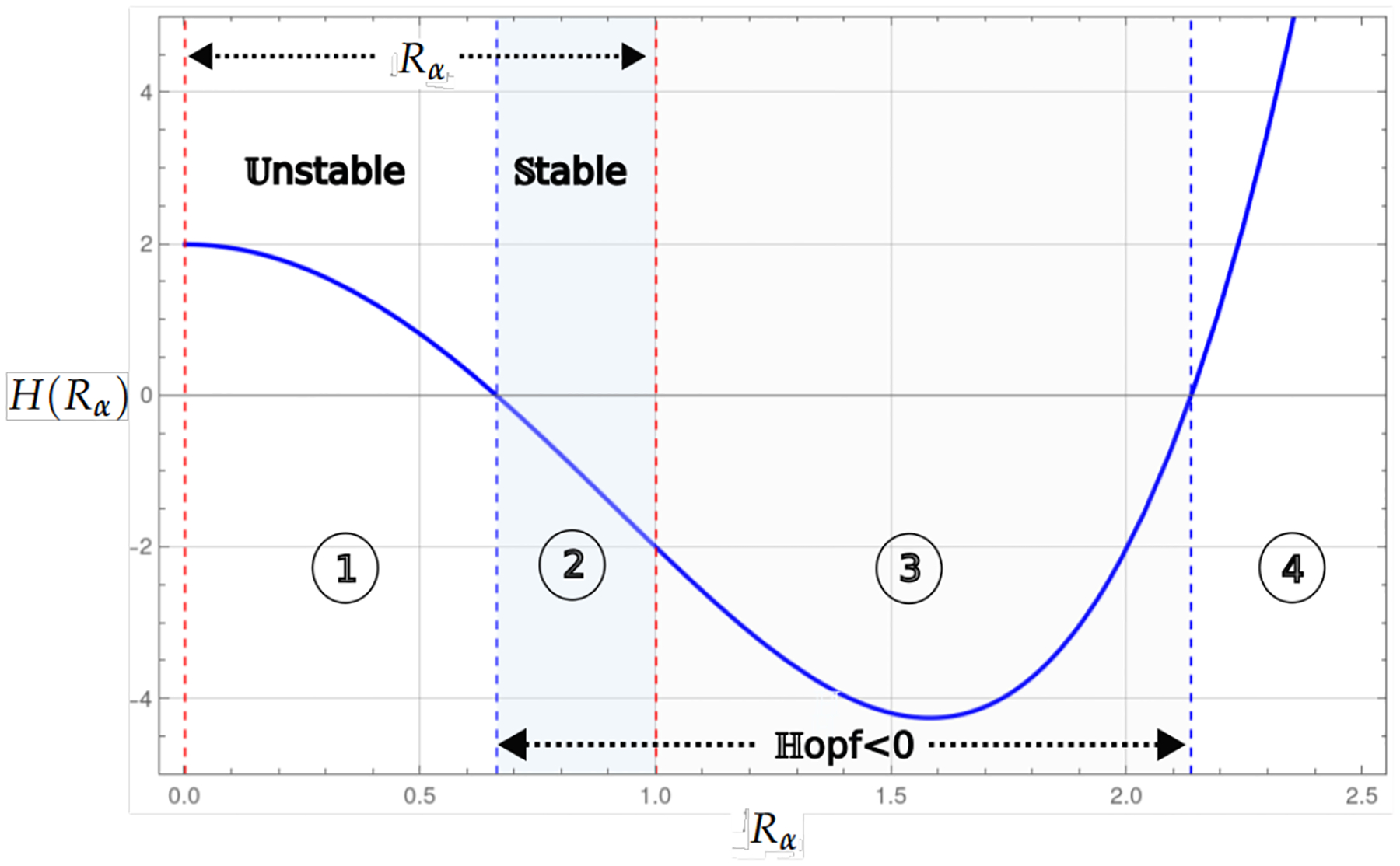
The bifurcation diagram corresponds to the model with linear growth and a strong Allee effect. The blue curve represents a projection of the Hopf hyperparametric set as a function of $R_{\alpha }$.

**Figure 3.**
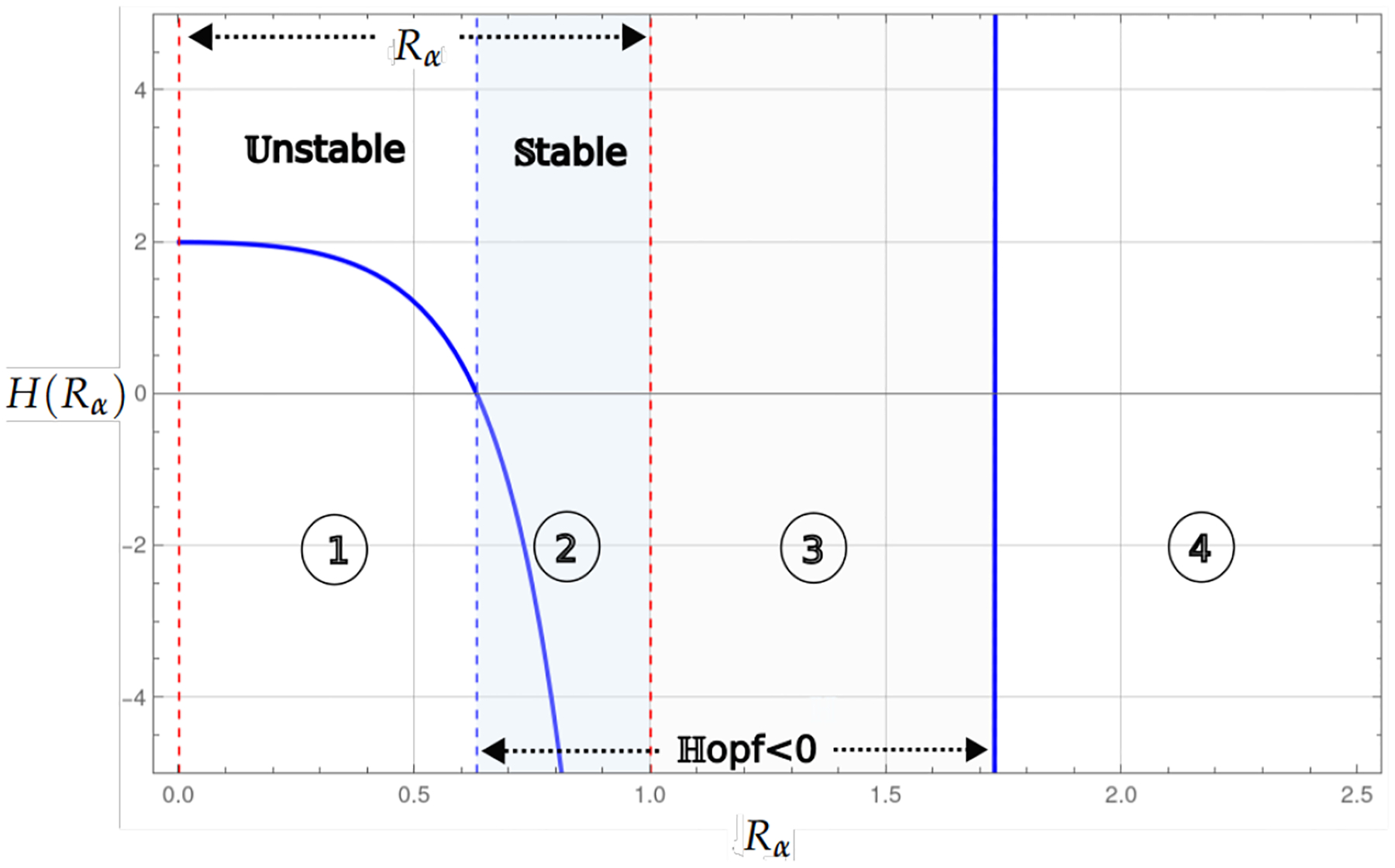
The bifurcation diagram corresponds to the model with linear growth and both weak and strong Allee effects (2). The blue curve represents a projection of the Hopf hyperparametric set (5) as a function of $R_{\alpha }$.

Theorem 1 and Proposition 1 show that for the values of the basic reproduction number $R_{\alpha }<1$, the existence of the endemic critical point, the system presents stability and instability; in instability, the population collapses to the trivial critical point, which is stable. On the other hand, stability may be associated with some level of cancer cells or with a cycle of co-existence through the Hopf bifurcation. Figures 2 and 3, shows this behavior, where the blue curve represents the projection of the hypersurface of Hopf bifurcations (5), by fixing the parameter values and varying the basic number reproductive term $R_{\alpha }$.

### 3.2. Linear Growth with Hyper Allee Effect

**Figure 4.**
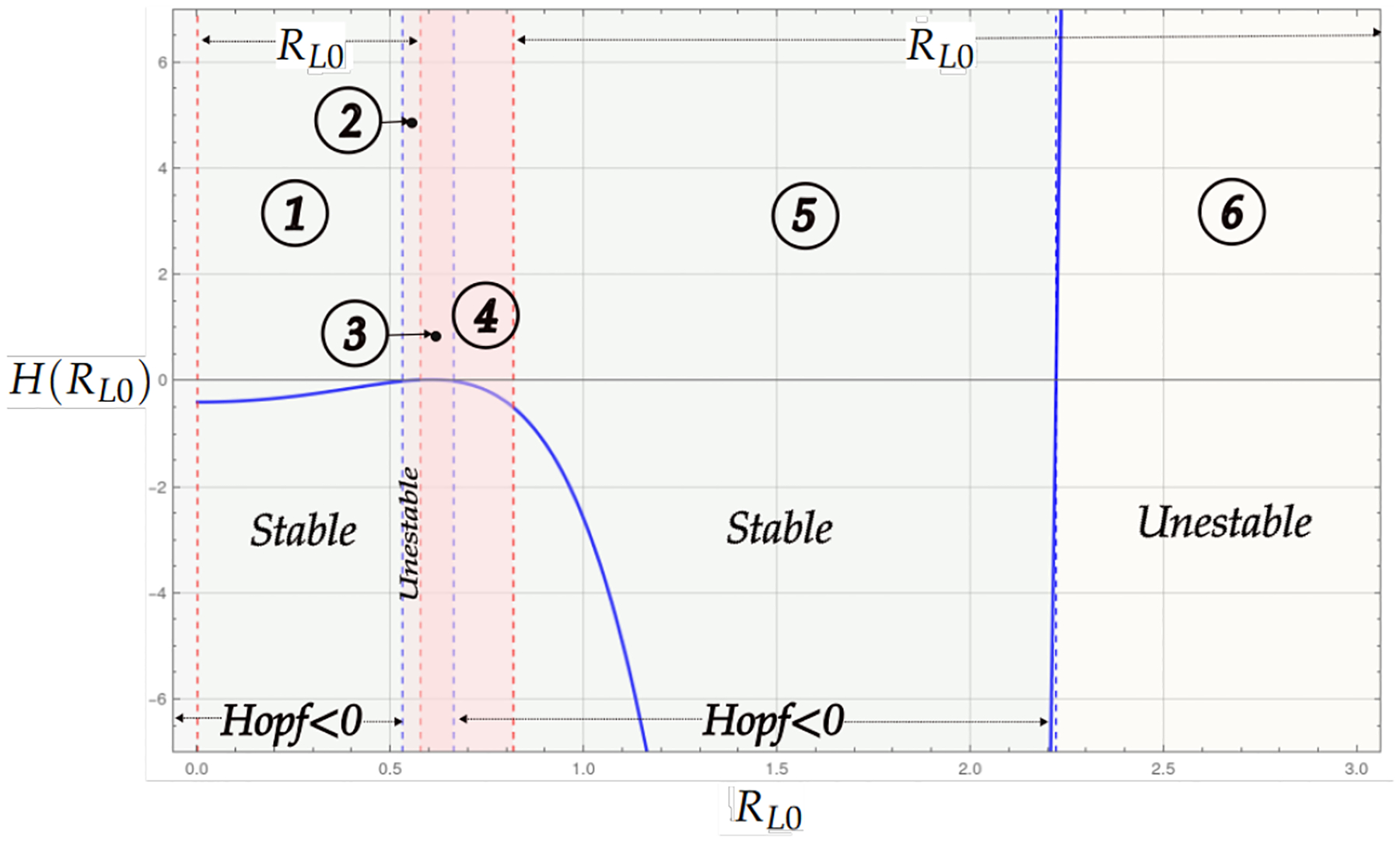
The bifurcation diagram corresponds to the model with linear growth and a hyper Allee effect (10). The blue curve represents a projection of the Hopf hyperparametric set (13) as a function of $R_{\alpha }$.

In the last part of this section, we address the case of the hyper Allee effect model in a linear growth context see Figure 4. The Allee effect, which describes a positive relationship between population density and growth rate, is fundamental to understanding complex dynamics such as virus-infected tumor cells. By integrating a hyper Allee effect into a linear growth model, we analyze the conditions that lead to endemic point stability. Let

(10) $$T^{\prime }=\lambda TA\left(T,\alpha_{1},\alpha_{2}\right)-\beta TV,\;I^{\prime }=\beta TV-\mu I,\;V^{\prime }=pI-\delta V,$$

where the term $A\left(T,\alpha_{1},\alpha_{2}\right)=\left(\alpha_{1}T-1\right)\left(\alpha_{2}T-1\right)$ represents the hyper Allee effect, with $\alpha_{1}<\alpha_{2}$. If we calculate the critical points, then we have the trivial (0, 0, 0), two critical points for free disease $\left(1/\alpha_{1},0,0\right)$, $\left(1/\alpha_{2},0,0\right)$, and one endemic critical point

$$P_{le_{H}}=\left(\frac{\delta \mu }{p\beta },\frac{\delta^{2}\lambda \mu \left(p\beta -\alpha_{1}\delta \mu \right)\left(p\beta -\alpha_{2}\delta \mu \right)}{p^{4}\beta^{4}},\frac{\delta \lambda \mu \left(p\beta -\alpha_{1}\delta \mu \right)\left(p\beta -\alpha_{2}\delta \mu \right)}{p^{3}\beta^{4}}\right).$$

Analogously to the calculation of $R_{\alpha }$ (3) thought $R_{T*}$, we perform the calculation for infection-free points $\left(1/\alpha_{1},0,0\right)$ and $\left(1/\alpha_{2},0,0\right)$, and we obtain

(11) $$R_{l\alpha_{1}}^{2}=\frac{p\beta }{\alpha_{1}\delta \mu }\;R_{l\alpha_{2}}^{2}=\frac{p\beta }{\alpha_{2}\delta \mu }$$

These points are now inversely related to the Allee hyper effect values $\alpha_{1}$ and $\alpha_{2}$. Here, we use the general structure of the basic reproduction number $R_{T^{*}}$ as the infectious threshold $R_{l0}$. If we take the following combination of parameters $R_{l0}^{2}=\frac{p\beta }{\delta \mu }$, then $R_{l\alpha_{1}}>R_{l\alpha_{2}}$, with $R_{l\alpha_{1}}^{2}=R_{l0}^{2}\left(1/\alpha_{1}\right)$ and $R_{l\alpha_{2}}^{2}=R_{l0}^{2}\left(1/\alpha_{2}\right)$. Then we have the critical point $P_{le_{H}}$ as

(12) $$P_{le_{H}}=\left(\frac{1}{R_{l0}^{2}},\frac{\left(R_{l0}^{2}-\alpha_{1}\right)\left(R_{l0}^{2}-\alpha_{2}\right)\lambda }{R_{l0}^{6}\mu },\frac{\left(R_{l0}^{2}-\alpha_{1}\right)\left(R_{l0}^{2}-\alpha_{2}\right)\lambda }{R_{l0}^{4}\beta }\right),$$

the existence conditions are $0<R_{l0}<\sqrt{\alpha_{1}}$ or $R_{l0}>\sqrt{\alpha_{2}}$. We will now study the Hopf bifurcation in this case.

**Theorem 2**. *Consider the system* (10). *The following set*

(13) $$H_{H}=\left\{\left(\lambda ,\varsigma_{l_{H_{1}}},\varsigma_{l_{H_{2}}},R_{l0}^{2}\right)|\varsigma_{l_{H_{1}}}\lambda -\varsigma_{l_{H_{2}}}^{2}=0\right\},$$

*contains the symmetric-saddles and Hopf bifurcations in the point* $P_{le_{H}}$ (12), *where*

(14) $$\varsigma_{l_{H_{1}}}=(\delta +\mu ){\left(R_{l0}^{2}\left(\alpha_{1}+\alpha_{2}\right)-2\alpha_{1}\alpha_{2}\right)}^{2}\;\varsigma_{l_{H_{2}}}=R_{l0}^{8}\delta \mu +R_{l0}^{4}\alpha_{1}\alpha_{2}(2\delta +\mu )(\delta +2\mu )\;-R_{l0}^{6}\left(\alpha_{1}+\alpha_{2}\right)\left(\delta^{2}+3\delta \mu +\mu^{2}\right)\;R_{l0}^{2}\neq \frac{2\alpha_{\alpha_{1}}\alpha_{2}}{\left(\alpha_{1}+\alpha_{2}\right)}$$

**Proof**. Taking the endemic point $P_{le_{H}}$ (12) and evaluating the Jacobian matrix A, in terms of parameters, then we calculate its characteristic polynomial similar to Equation (7), and we calculate the parameters $a_{1},a_{2}$, and $a_{3}$ as in the Hurwitz matrix H. We obtain the set of parameters (13), when looking for conditions where $\left(a_{1}a_{2}-a_{3}\right)=0$. □

**Proposition 2**. *Let* $0<R_{l0}^{2}<\alpha_{1}$ *or* $R_{l0}^{2}>\alpha_{2}$ *and* $\varsigma_{l_{H_{1}}}\lambda <\varsigma_{l_{H_{2}}}^{2}$, *and then the endemic critical point* $P_{le_{H}}$ (12) *of a system* (10), *is locally asymptotically stable*.

**Proof**. Following the proof of the weak and strong Allee case, using the Hopf bifurcation set and a parameter ϵ, we obtain the coefficients of the Hurwitz matrix, denoted as $a_{1},a_{2}$, and $a_{3}$. Also, we considers the symmetric-saddle and Hopf bifurcations from (13), such that

(15) $$\lambda ≔\epsilon \frac{\varsigma_{l_{H_{2}}}^{2}}{\varsigma_{l_{H_{1}}}},$$

if we evaluate in $\left(a_{1}a_{2}-a_{3}\right)$, then we obtain

(16) $$\left(a_{1}a_{2}-a_{3}\right)=\epsilon (\epsilon -1)\frac{\varsigma_{l_{H_{2}}}^{2}}{\varsigma_{l_{H_{1}}}}.$$

The critical point $P_{le_{2}}$ (12) is locally asymptotically stable under $0<R_{l0}^{2}<\alpha_{1}$ or $R_{l0}^{2}>\alpha_{2}$ and $\varsigma_{l_{H_{1}}}\lambda <\epsilon \varsigma_{l_{H_{2}}}^{2}$, with ϵ>1. □

Theorem 2 and Proposition 2 analyze the case in which the endemic critical point is stable, and its existence is divided into two regions depending on $R_{l0}$. If $R_{l0}<\sqrt{\alpha_{1}}$, the endemic critical point can be stable or unstable. Stability occurs at a certain level of infected cancer cells or through limit cycles caused by the presence of a Hopf bifurcation. In the case where $R_{l0}>\sqrt{\alpha_{2}}$, the region of stability and instability is determined by the Hopf bifurcation, where, in a neighborhood, infected tumor cells coexist in a limit cycle. The bifurcation diagram shows larger instability regions for $R_{l0}>\sqrt{\alpha_{2}}$. This suggests a higher number of infected cancer cells and, eventually, tumor shrinkage in the presence of a high hyper Allee value $\alpha_{2}$.

This section examined the linear population growth model incorporating weak, strong, and weak with their combinations and hyper Allee effects. This analysis shows how these effects significantly influence population dynamics and stability of virus-infected tumor cells. The bifurcation diagrams presented in Section 5 illustrate the different cases and critical points that emerge under different configurations of the Allee effect parameters. The following section will review the logistic growth case and explore how the Allee effects affect this model, which will allow us to compare the dynamics obtained under different population growth assumptions, enriching our understanding of the conditions that favor stability and, if so, its eventual application in the effective control of tumor populations.

## 4. Logistic Growth Model

This section will consider the base model (1) with logistic growth. Following the approach of the previous Section 3, we analyze only representative cases of the model dynamics. For example, in that section, we examined the model with linear growth under the weak Allee effect since its dynamics do not show significant differences compared to the case without such an effect. Similarly, the case of the model that simultaneously includes weak and strong Allee effects is similar to the case where only a strong Allee effect is considered. To verify these observations, one can perform the corresponding calculations or consult the associated typical diagrams, as shown in Section 5 on bifurcation diagrams.

### 4.1. Logistic Growth Without Allee Effect

In order to enrich our analysis and explore the dynamics of the base system (1) more fully, we added the logistic growth model. This model incorporates the carrying capacity and is a way to model the systems more realistically compared to linear growth, where an unbounded population is considered, which allows us to study the population dynamics of tumor cells under a limited number that depends on each patient, in this case not including the Allee effect in conjunction with this carrying capacity because we want to see the dynamic without the Allee effect. In the following, we present the system and the parameters for its analysis.

(17) $$T^{\prime }=\lambda T(1-bT)-\beta TV,\;I^{\prime }=\beta TV-\mu I,\;V^{\prime }=pI-\delta V$$

where b represents the carrying capacity of the system, that is, the maximum limit of the tumor population. If we calculate the critical points here, we obtain the trivial one (0, 0, 0), the disease-free equilibrium point $P_{0}=\left(T_{0},I_{0},V_{0}\right)=\left(\frac{1}{b},0,0\right)$, and the endemic point

(18) $$P_{ge}=\left(\frac{\delta \mu }{p\beta },\frac{\delta \lambda (p\beta -b\delta \mu )}{(p\beta {)}^{2}},\frac{\lambda (p\beta -b\delta \mu )}{p\beta^{2}}\right).$$

As in system (1), system (17) is equivalent to those of an SEIR epidemiological model with the assumption of a constant population size. This equivalence implies that the dynamics of these systems are also similar, and results known for the SEIR model can be straightforwardly extended to the branch of the system of (1) like the basic reproductive number, similar to that founded in $R_{\alpha }$ in the previous section. The basic reproductive number $R_{0}$ is the expected number of secondary cases produced in a susceptible population by a typical ineffective individual when the tumor cell is infectious. In this model (17), $R_{0}$ denotes the average number of new tumor cells infected by a virion during its lifetime when placed in a population of fully susceptible tumor cells.

As for ${\left.R_{T^{*}}\right|}_{T^{*}=\frac{1}{b}}$ or $R_{\alpha }$ (3), using the next-generation matrix method by [43], we obtain

(19) $$R_{0}^{2}=\frac{p\beta }{b\delta \mu },$$

If the virus’s basic reproductive number is smaller than 1, then at the beginning of the infection, each virus-infected tumor cell produces, on average, less than one newly infected tumor cell. Therefore, the infection cannot spread and the system returns to an uninfected state. If $R_{0}>1$, then initially each virus-infected tumor cell produces, on average, more than one newly infected cell.

Taking into account the endemic equilibrium point $P_{ge}$ and the basic reproductive number (19), we represent the state in which the virus is present as

$$T_{ge}=\frac{1}{bR_{0}^{2}},$$

$$I_{ge}=\left(R_{0}^{2}-1\right)\frac{\lambda }{b\mu R_{0}^{4}},$$

$$V_{ge}=\left(R_{0}^{2}-1\right)\frac{\lambda }{\beta R_{0}^{2}}.$$

The conditions of existence of this point are given by $1<R_{0}^{2}$. On the other hand, the stability of the critical point free of infection $P_{0}=\left(\frac{1}{b},0,0\right)$ is as follows.

**Proposition 3**. *For the critical point* $P_{0}$,

1. *If* $R_{0}<1$, *then the three eigenvalues are real and negative*.
2. *If* $R_{0}=1$, *then two eigenvalues are real and negative, and one equals zero*.
3. *if* $R_{0}>1$, *then two eigenvalues are real and negative and one is real and positive*.

**Proof**. First, we take the linearization of the system at $P_{0}$ as

$$A_{0}=\left(\begin{array}{ccc} -\lambda & 0 & -\frac{\beta }{b}\\ 0 & -\mu & \frac{\beta }{b}\\ 0 & p & -\delta \end{array}\right)$$

The characteristic polynomial is

(20) $$0=\left|\begin{array}{ccc} \left(-\lambda -\chi \right) & 0 & -\frac{\beta }{b}\\ 0 & -(\mu +\chi ) & \frac{\beta }{b}\\ 0 & p & -(\delta +\chi ) \end{array}\right|\;=-(\lambda +\chi )\left((\mu +\chi )(\delta +\chi )-\frac{p\beta }{b}\right)\;=-(\lambda +\chi )\left(\chi^{2}+(\delta +\mu )\chi +\delta \mu -\frac{p\beta }{b}\right)$$

The second factor can be expressed as

$$Q=\chi^{2}+(\delta +\mu )\chi +\delta \mu -\frac{p\beta }{b}\;=\chi^{2}+(\delta +\mu )\chi +\delta \mu \left(1-\frac{p\beta }{\delta \mu b}\right)\;=\chi^{2}+(\delta +\mu )\chi +\delta \mu \left(1-R_{0}^{2}\right)$$

Then, the roots are

(21) $$\chi =\frac{1}{2}\left(-(\delta +\mu )\pm \sqrt{(\delta +\mu {)}^{2}-4\left(1-R_{0}^{2}\right)\delta \mu }\right)$$

The equivalent expressions for the discriminant are

$$\Delta =(\delta +\mu {)}^{2}-4\left(1-R_{0}^{2}\right)\delta \mu \;=\delta^{2}+2\delta \mu +\mu^{2}-4\delta \mu \left(1-R_{0}^{2}\right)\;=(\delta -\mu {)}^{2}+4R_{0}^{2}\delta \mu$$

Since the discriminant Δ>0, for any $R_{0}>0$ and positive parameters δ,μ>0, all roots are real. For $R_{0}<1$, the discrimimant $\Delta <(\delta +\mu {)}^{2}$; therefore, both roots are negative for and bα<1. If $R_{0}>1$, then $\Delta >(\kappa +\delta {)}^{2}$ and one of the roots in (21) is positive. □

In the following result, we will analyze the Hopf bifurcations set as a function of admissible parameters of the system (17) at the critical point $P_{ge}$ (18).

**Theorem 3**. *Consider the system of autonomous equations that model the dynamics of the tumor cell population, given by* (17). *The following set*

(22) $$H=\left\{\left(\lambda ,\Phi_{01},\Phi_{02},R_{0}^{2}\right)|\Phi_{01}\lambda -\Phi_{02}^{2}=0\right\},$$

*contains the symmetric-saddles and Hopf bifurcations in the point* $P_{ge}$, *where*

(23) $$\Phi_{01}=(\delta +\mu )\;\Phi_{02}=\left(\left(\delta^{2}+\mu^{2}\right)-\left(R_{0}^{2}-3\right)\delta \mu \right)R_{0}^{2}.$$

**Proof**. Taking the endemic point $P_{ge}$ and evaluating in the Jacobian matrix A, we calculate its characteristic polynomial, to calculate the coefficients of Hurwitz matrix as follows:

$$a_{1}=(\delta +\mu )+\frac{\lambda }{R_{0}^{2}}\;a_{2}=(\delta +\mu )\frac{\lambda }{R_{0}^{2}}\;a_{3}=\left(R_{0}^{2}-1\right)\frac{\delta \lambda \mu }{R_{0}^{2}}$$

All coefficients are positive for $R_{0}^{2}>1$. By finding the condition for $\left(a_{1}a_{2}-a_{3}\right)=0$, we obtain the set of parameters (22). □

The Hopf bifurcation represents a transition to stability in the system. This bifurcation occurs when we move one key parameter and obtain a pair of complex conjugate eigenvalues, which can be the basic reproductive number $R_{0}^{2}$. If we take (7), with conditions $a_{0}=1$ and $a_{1}\cdot a_{2}=a_{3}$, then we obtain one real eigenvalue given by $\psi =-a_{1}$ and two conjugate complexes of eigenvalues $\psi =\pm i\sqrt{a_{2}}$, with $a_{2}>0$. When $a_{2}$ is the change in sing, then complex conjugate eigenvalues cross the imaginary axis and appear in the Hopf bifurcation; in this case, the change in sing is given by (22), and if $a_{2}<0$, then we have the symmetric-saddles.

These eigenvalues show a transition in the system’s dynamics, in which the solutions change from unstable to oscillatory behavior; under small perturbations, the system could deviate from the cycle and move towards a different solution or a higher instability. In Section 5, we present the bifurcation diagram corresponding to the Theorem 3, which graphically illustrates some equilibrium phases in the system (17).

Analyzing the endemic equilibrium point is essential to identify regions in terms of parameters where dynamic behaviors such as stability, periodic oscillations, or critical transitions of sign change points are present. In the following, we present an analysis that characterizes this point.

**Proposition 4**. *Let* $1<R_{0}^{2}$ *and* $\Phi_{01}\lambda <\Phi_{02}^{2}$*, and then the endemic critical point* $P_{ge}$ *of the system* (17) *is locally asymptotically stable*.

**Proof**. As in the proof of Theorem 3, we calculate the coefficients of characteristic polynomial $a_{1},a_{2},a_{3}$ which are all positive for the existence condition of endemic critical point $P_{ge}$. Following the stability proof in the previous section, we define the parameter λ in terms of ϵ, but with $\Phi_{01}$ and $\Phi_{02}$. If we evaluate in $\left(a_{1}a_{2}-a_{3}\right)$, we have

$$\left(a_{1}a_{2}-a_{3}\right)=(\epsilon -1)\epsilon \frac{{\left(\delta^{2}+\mu^{2}-\left(R_{0}^{2}-3\right)\delta \mu \right)}^{2}}{\delta +\mu }.$$

Then, for ϵ>1, we obtain $\left(a_{1}a_{2}-a_{3}\right)>0$ and the critical point $P_{ge}$ is locally asymptotically stable. □

**Figure 5.**
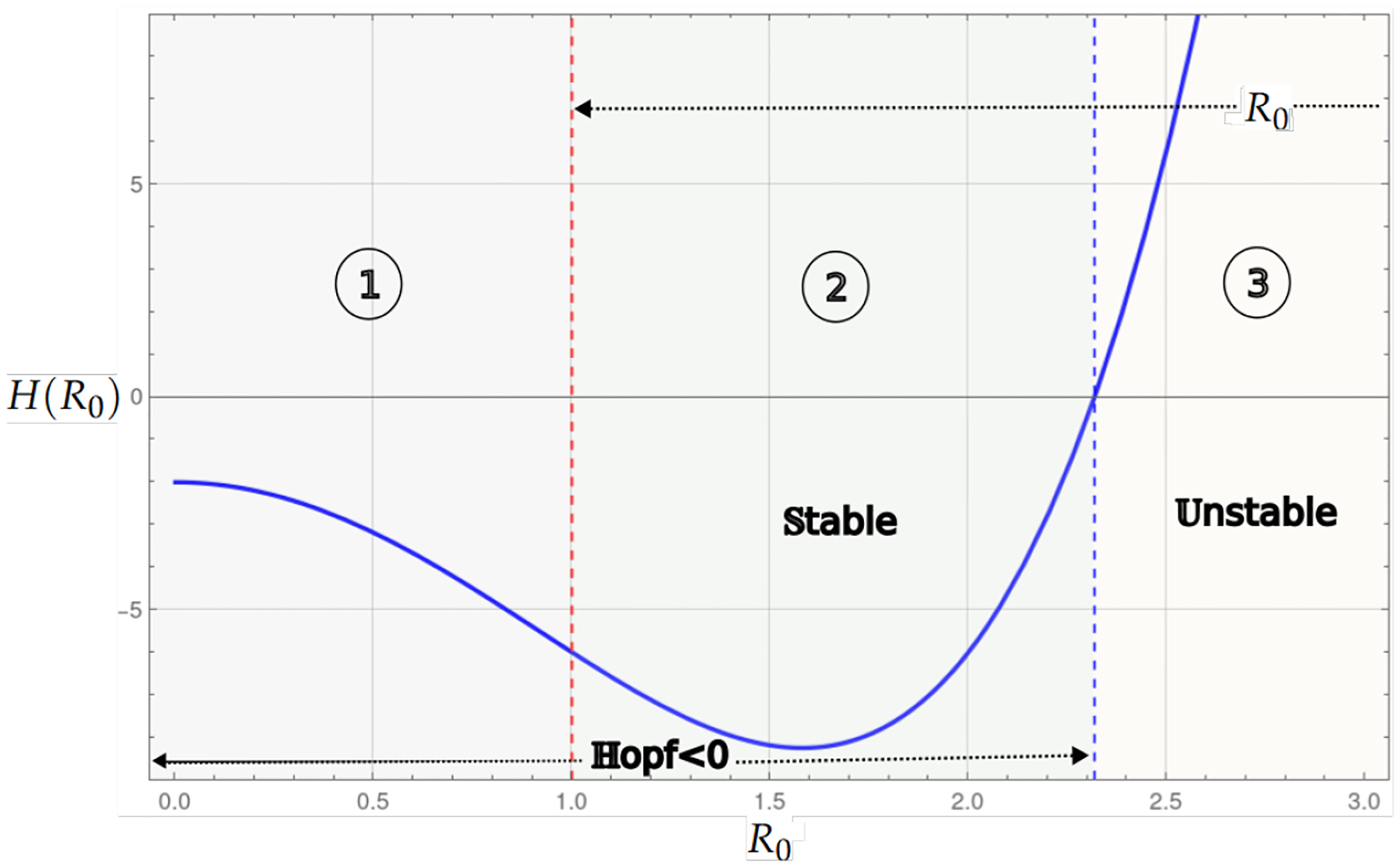
The bifurcation diagram corresponds to the model with logistic growth and no Allee effect (17). The blue curve represents a projection of the Hopf hyperparametric set (22) as a function of $R_{0}$.

The summary of the dynamics of the logistic growth case without the Allee effect is presented in the bifurcation diagram in Figure 5. We observed the condition of existence $R_{0}>1$ of the endemic critical point $P_{ge}$ (18) and the Hopf curve, which is a threshold that, in addition to determining the stability of the critical point, can exhibit limit cycles in a neighborhood of parameters. In instability, a trajectory away from the unstable endemic equilibrium may tend to the trivial stable critical point, indicating the collapse of the system, or to the stable variety of the infection-free saddle point $P_{0}=(1/b,0,0)$, indicating the propagation of cancer cells.

### 4.2. Logistic Growth with Strong Allee Effect

This model incorporates the strong Allee effect parameter α. We present the system for its analysis in the following.

(24) $$T^{\prime }=\lambda T(1-bT)(T-\alpha )-\beta TV,\;I^{\prime }=\beta TV-\mu I,\;V^{\prime }=pI-\delta V$$

where b represents the carrying capacity of the system, and α represents the strong Allee effect, which describes the decrease in population growth rate when tumor cell density is below a critical threshold, where we want to explore its influence on the dynamics of the system. If we calculate the critical points here, we obtain the trivial one (0, 0, 0), the disease-free equilibrium point $P_{0s}=\left(\frac{1}{b},0,0\right)$ in terms of the braking capacity, the disease-free equilibrium point $P_{\alpha }=(\alpha ,0,0)$ in terms of the strong Allee effect, and the endemic point

$$P_{gse}=\left(\frac{\delta \mu }{p\beta },-\frac{\delta \lambda (p\alpha \beta -\delta \mu )(p\beta -b\delta \mu )}{(p\beta {)}^{3}},-\frac{\delta \lambda (p\alpha \beta -\delta \mu )(p\beta -b\delta \mu )}{p^{2}\beta 3}\right).$$

If we substitute the basic reproductive number $R_{0}^{2}$, from (19), because it is the same in the system (24), we obtain

$$P_{gse}=\left(\frac{1}{bR_{0}^{2}},-\left(R_{0}^{2}-1\right)\left(\alpha bR_{0}^{2}-1\right)\frac{\lambda }{b^{2}\mu R_{0}^{6}},-\left(R_{0}^{2}-1\right)\left(\alpha bR_{0}^{2}-1\right)\frac{\lambda }{b\beta R_{0}^{4}}\right),$$

in terms of $R_{0}$. Considering the critical point $P_{\alpha }=(\alpha ,0,0)$, we also introduce the term $R_{\alpha }$ from (3) to represent the critical point $P_{gse}$ as a function of the two basic reproduction numbers, $R_{0}$ and $R_{\alpha }$, as follows.


(25) $$P_{gse}=\left(\frac{1}{bR_{0}^{2}},-\left(R_{0}^{2}-1\right)\left(R_{\alpha }^{2}-1\right)\frac{\lambda }{b^{2}\mu R_{0}^{6}},-\left(R_{0}^{2}-1\right)\left(R_{\alpha }^{2}-1\right)\frac{\lambda }{b\beta R_{0}^{4}}\right).$$

The conditions of existence of this point are given by $R_{\alpha }^{2}<1<R_{0}^{2}$. We observe that the basic reproductive numbers have the following relation

(26) $$R_{\alpha }^{2}=R_{0}^{2}b\alpha .$$

Note that the stability analysis of the disease-free point $P_{0s}$ is analogous to that performed in the previous system. However, the associated characteristic polynomial presents a modified expression, which is detailed below.


$$A_{0}=\left(\begin{array}{ccc} \left(\alpha -\frac{1}{b}\right)\lambda & 0 & -\frac{\beta }{b}\\ 0 & -\mu & \frac{\beta }{b}\\ 0 & p & -\delta \end{array}\right)$$

The characteristic polynomial is

(27) $$0=\left|\begin{array}{ccc} \left(\left(\alpha -\frac{1}{b}\right)\lambda -\chi \right) & 0 & -\frac{\beta }{b}\\ 0 & -(\mu +\chi ) & \frac{\beta }{b}\\ 0 & p\gamma & -(\delta +\chi ) \end{array}\right|\;=\left(\left(\alpha -\frac{1}{b}\right)\lambda -\chi \right)\left((\mu +\chi )(\delta +\chi )-\frac{p\beta }{b}\right)\;=\left(\left(\alpha -\frac{1}{b}\right)\lambda -\chi \right)\left(\chi^{2}+(\delta +\mu )\chi +\delta \mu \left(1-R_{0}^{2}\right)\right)$$

So, we have a Corollary from Proposition 3.

**Corollary 1**. *For the critical point* $P_{0}$,

1. *If* $R_{0}<1$, *then the three eigenvalues are real and negative*.
2. *If* $R_{0}=1$, *then two eigenvalues are real and negative, and one equals zero*.
3. *if* $R_{0}>1$, *then two eigenvalues are real and negative and one is real and positive*.

In the following result, we will analyze the Hopf bifurcations set as a function of admissible parameters of the system (24) at the critical point $P_{gse}$ (25).

**Theorem 4**. *Consider the system given by* (24). *The following set*

(28) $$H=\left\{\left(\lambda ,\Phi_{s1},\Phi_{s2},R_{0}^{2}\right)|\Phi_{s1}\lambda -\Phi_{s2}^{2}=0\right\},$$

*contains the symmetric-saddles and Hopf bifurcations in the point* $P_{gse}$, *where*

(29) $$\Phi_{s1}=\Delta_{R_{0,\alpha }}^{2}(\delta +\mu )\;\Phi_{s2}=\delta \mu \left(R_{0}^{2}\left(\alpha b\left(R_{0}^{2}-3\right)-3\right)+5\right)-\Delta_{R_{0,\alpha }}\left(\delta^{2}+\mu^{2}\right)\;\Delta_{R_{0,\alpha }}≔R_{0}^{2}(1+\alpha b)-2.$$

**Proof**. Take the endemic point $P_{gse}$ and evaluate the Jacobian matrix A to look for conditions where the (TrASimA) minus the determinant DetA are zero. Using the resulting polynomials ${\text{Pol}}_{0}$ as in [45,46], we obtain the set of parameters (28). □

The predicted result confirms the existence of a Hopf bifurcation in the system (24). This result indicates the emergence of periodic orbits near the analyzed equilibrium point, which introduces oscillatory dynamics into the model. Next, we explore the stability of the equilibrium point, which will allow us to identify the conditions under which stability exists.

**Proposition 5**. *Let* $1<R_{0}^{2}<2/(1+b\alpha )$ *and* $\Phi_{s1}\lambda <\Phi_{s2}^{2}$, *and then the endemic critical point* $P_{\text{gse}}$ *of a system* (24) *is locally asymptotically stable*.

**Proof**. The linearization of the system (24) at $P_{gse}$ is given by

$$A_{1}=\left(\begin{array}{ccc} \frac{\lambda \left(\left(R_{0}^{2}-1\right)+\left(R_{\alpha }^{2}-1\right)\right)}{bR_{0}^{4}} & 0 & -\frac{\beta }{bR_{0}^{2}}\\ -\frac{\lambda \left(R_{0}^{2}-1\right)\left(R_{\alpha }^{2}-1\right)}{bR_{0}^{4}} & -\mu & \frac{\beta }{bR_{0}^{2}}\\ 0 & p & -\delta \end{array}\right)$$

The characteristic polynomial associated with $A_{1}$ is

(30) $$p(\psi )=a_{0}\psi^{3}+a_{1}\psi^{2}+a_{2}\psi +a_{3},$$

in terms of the trace, the sum of all second-order diagonal minors of $A_{1}$ denoted by ${\text{SimA}}_{1}$, and the determinant of the matrix $A_{1}$, where $a_{0}=1,a_{1}=-{\text{TrA}}_{1},a_{2}=\text{Sim}A_{1}$, and $a_{3}=-DetA_{1}$. Specifically

$$a_{0}=1,\;a_{1}=(\delta +\mu )-\lambda \frac{\left(R_{0}^{2}-1\right)+\left(R_{\alpha }^{2}-1\right)}{bR_{0}^{4}},\;a_{2}=-\lambda (\delta +\mu )\frac{\left(R_{0}^{2}-1\right)+\left(R_{\alpha }^{2}-1\right)}{bR_{0}^{4}},\;a_{3}=(\lambda \delta \mu )\frac{\left(R_{0}^{2}-1\right)\left(1-R_{\alpha }^{2}\right)}{bR_{0}^{4}}.$$

The Hurwitz matrix associated with p(ψ) is given by

$$H(p)=\left(\begin{array}{ccc} a_{1} & a_{3} & 0\\ a_{0} & a_{2} & 0\\ 0 & a_{1} & a_{3} \end{array}\right)$$

according to the Hurwitz stability [44], $P_{gse}$ is locally asymptotically stable if $a_{1},a_{2},a_{3}>0$ and $a_{1}a_{2}-a_{3}>0$. Under the conditions of the existence of the endemic point $P_{gse},R_{\alpha }^{2}<1<R_{0}^{2}$ or equivalently $1<R_{0}^{2}<1/(b\alpha )$. It follows that

$$\Delta_{R_{0,\alpha }}≔\left(R_{0}^{2}-1\right)+\left(R_{\alpha }^{2}-1\right)\;=R_{0}^{2}(1+b\alpha )-2<0,\;\text{or}\;1<R_{0}^{2}<\frac{2}{1+b\alpha }.$$

So $a_{1}>0$ with $1<R_{0}^{2}<2/(1+b\alpha )$ or when $R_{0}^{2}\geq 2/(1+b\alpha )$ with $(\delta +\mu )>\lambda \Delta_{R_{0,\alpha }}$. Also, the coefficient $a_{2}$ is positive with $1<R_{0}^{2}<2/(1+b\alpha )$. It holds that $a_{3}>0$ for $R_{\alpha }^{2}<1<R_{0}^{2}$ or equivalently $1<R_{0}^{2}<1/(b\alpha )$. As we can see,

$$-(\delta +\mu )\Delta_{R_{0,\alpha }}\left((\delta +\mu )-\frac{\lambda \Delta_{R_{0,\alpha }}}{bR_{0}^{2}}\right)>0,$$

in $1<R_{0}^{2}<2/(1+b\alpha )$ and

$$\left(R_{0}^{2}-1\right)\left(1-R_{\alpha }^{2}\right)>0,$$

in $1<R_{0}^{2}<1/(b\alpha )$, then

$$\left[-(\delta +\mu )\Delta_{R_{0,\alpha }}\left((\delta +\mu )-\frac{\lambda \Delta_{R_{0,\alpha }}}{bR_{0}^{2}}\right)+\left(R_{0}^{2}-1\right)\left(1-R_{\alpha }^{2}\right)\delta \mu \right]>0,$$

for $1<R_{0}^{2}<2/(1+b\alpha )$. Which implies that $a_{1}a_{2}-a_{3}>0$. On the other hand, taking (28) we have

$$\lambda =\epsilon \frac{\Phi_{s2}^{2}}{\Phi_{s1}},$$

with ϵ>0 and $\Phi_{s1}>0$. When we evaluate the last expression in $\left(a_{1}a_{2}-a_{3}\right)$ we obtain

$$\left(a_{1}a_{2}-a_{3}\right)=(\epsilon -1)\epsilon \frac{\Phi_{s2}^{2}}{\Phi_{s1}}.$$

In this case, if ϵ=1, then we have $a_{1}a_{2}-a_{3}=0$, which is equivalent to the Hopf condition, but if ϵ>1, also, $a_{1}a_{2}-a_{3}>0$. For all, $P_{gse}$ is stable when $1<R_{0}^{2}<2/(1+b\alpha )$ and $\lambda \Phi_{s1}<\Phi_{s2}^{2}$. □

**Figure 6.**
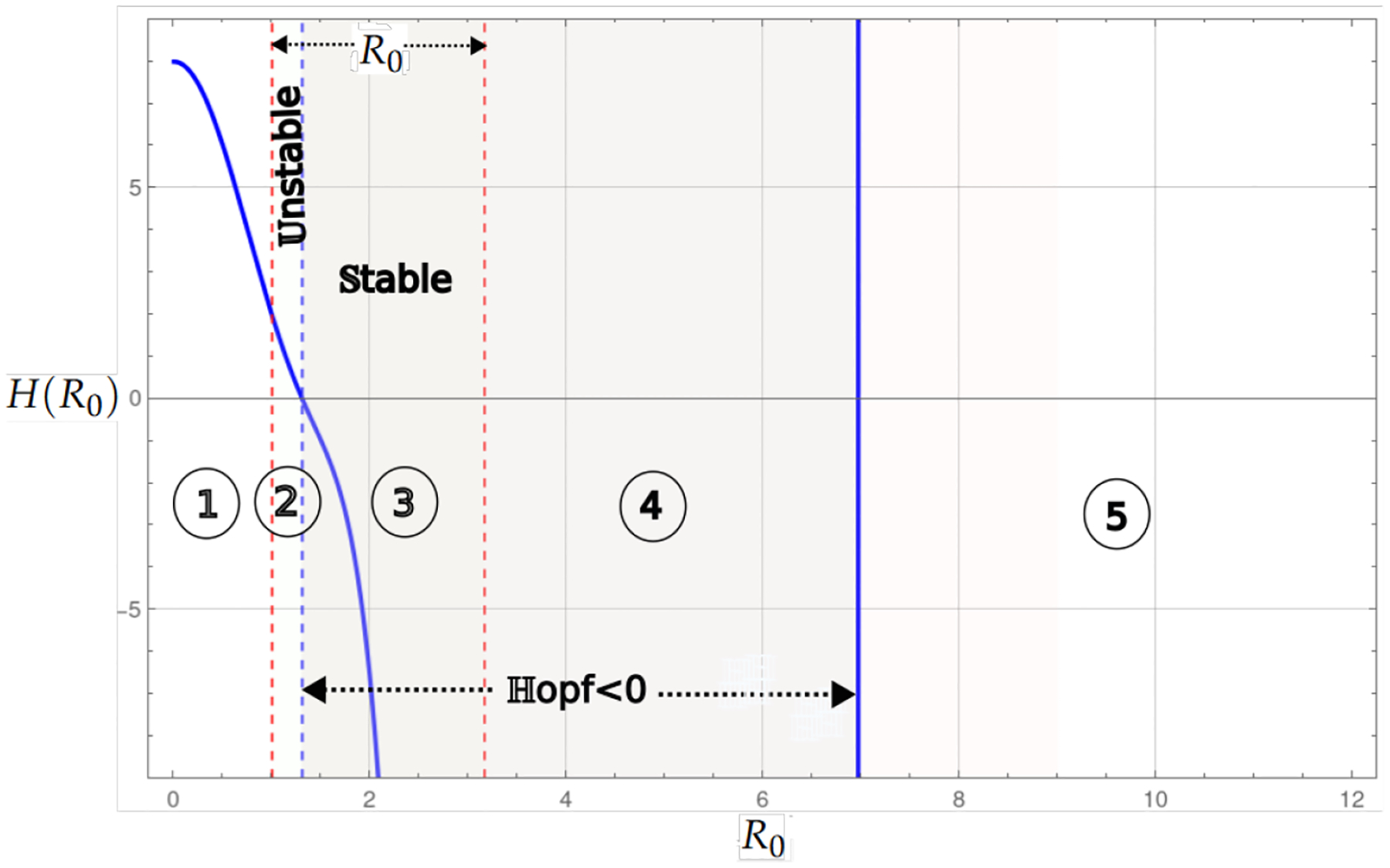
The bifurcation diagram corresponds to the model with logistic growth and strong Allee effect (24). The blue curve represents a projection of the Hopf hyperparametric set (28) as a function of $R_{0}$.

The bifurcation diagram in Figure 6 shows the dynamics when there is logistic growth and a strong Allee effect. The interval of existence of the critical point $P_{gse}$ is determined by the red dotted vertical lines determined by $1<R_{0}^{2}<2/(1+b\alpha )$, and in this case the point can be stable or unstable bounded by the Hopf bifurcation (blue curve). There may be endemic equilibrium or latent cancer, and there may also be escape from cancer; in this case, the infection-free point is $P_{0s}$.

### 4.3. Logistic Growth with Hyper Allee Effect

In this model, the population experiences two thresholds: one below, which tends to extinction, and another above, which again tends to extinction. The growth rate becomes negative at very low population sizes and high population sizes, with a positive growth rate only within a specific intermediate range. We present a mathematical representation with the hyper Allee effect by modifying the logistic growth model with additional terms that create a region of bi-stability.

(31) $$T^{\prime }=\lambda T(1-bT)\left(\alpha_{1}T-1\right)\left(\alpha_{2}T-1\right)-\beta TV,\;I^{\prime }=\beta TV-\mu I,\;V^{\prime }=pI-\delta V,$$

where b is the carrying capacity $\alpha_{1}$ and $\alpha_{2}$ are the two critical threshold with $\left(b<\alpha_{1}<\alpha_{2}\right)$. The other parameters remain the same as in the base system (1).

As in the previous case with the strong Allee effect, we take the basic reproductive number from (19) for the critical point $\left(\frac{1}{b},0,0\right)$. For the critical point $P_{\alpha_{1}}=\left(\frac{1}{\alpha_{1}},0,0\right)$, we obtain $R_{\alpha_{1}}=\sqrt{\frac{p\beta }{\alpha_{1}\delta \mu }}$. In addition to the critical point $P_{\alpha_{2}}=\left(\frac{1}{\alpha_{2}},0,0\right)$, we obtain $R_{\alpha_{2}}=\sqrt{\frac{p\beta }{\alpha_{2}\delta \mu }}$, which are expressions similar to (11). Then,

(32) $$R_{\alpha_{1}}^{2}=R_{0}^{2}\frac{b}{\alpha_{1}},\;R_{\alpha_{2}}^{2}=R_{0}^{2}\frac{b}{\alpha_{2}}.$$

Note that for $b<\alpha_{1}<\alpha_{2}$, then $R_{\alpha_{2}}^{2}<R_{\alpha_{1}}^{2}<R_{0}^{2}$.

In this analysis, we first focus on the stability of the disease-free critical points: $P_{b}=(1/b,0,0)$, $\left(1/\alpha_{1},0,0\right)$, and $\left(1/\alpha_{2},0,0\right)$. These points represent states in which infected tumor cells and virions are absent while infection-free tumor cells remain in equilibrium. While b denotes the system’s carrying capacity, the $\alpha_{1}$ and $\alpha_{2}$ correspond to hyper Allee effects on tumor cell dynamics.

Stability analysis of these points is essential to understand how system parameters, particularly the carrying capacity and Allee effects, influence the eradication or persistence of viral infection. We then performed a linear stability study, evaluating the zero solutions of the characteristic polynomial associated with these points.

**Proposition 6**. *For the following critical points* $P_{b},P_{\alpha_{1}}$, *and* $P_{\alpha_{2}}$, *we have the following*.

1. *If* $R_{0}<1$ *or* $R_{\alpha_{2}}<1$, *then the three eigenvalues are real negative. If* $R_{\alpha_{1}}<1$, *then two eigenvalues are negative real, and one is positive real*.
2. *If* $R_{0}=1$ *or* $R_{\alpha_{2}}=1$, *then two eigenvalues are negative real, and one equals zero. If* $R_{\alpha_{1}}=1$, *then one eigenvalue is positive, one negative, and one zero*.
3. *If* $R_{0}>1$ *or* $R_{\alpha_{2}}>1$, *then two eigenvalues are negative real, and one is positive real. If* $R_{\alpha_{1}}>1$, *then two eigenvalues are real positive and one is real and negative*.

**Proof**. The discriminant for each point $P_{0},P_{\alpha_{1}},P_{\alpha_{2}}$, with its corresponding third eigenvalue, is as follows.

$$\Delta_{b}=(\delta -\mu {)}^{2}+4R_{0}^{2}\delta \mu ,\;-\frac{\left(b-\alpha_{1}\right)\left(b-\alpha_{2}\right)}{b}<0,\;\Delta_{\alpha_{1}}=(\delta -\mu {)}^{2}+4R_{\alpha_{1}}^{2}\delta \mu ,\;\frac{\left(b-\alpha_{1}\right)\left(\alpha_{1}-\alpha_{2}\right)}{\alpha_{1}^{2}}>0,\;\Delta_{\alpha_{2}}=(\delta -\mu {)}^{2}+4R_{\alpha_{2}}^{2}\delta \mu ,\;\frac{\left(b-\alpha_{2}\right)\left(\alpha_{2}-\alpha_{1}\right)}{\alpha_{1}^{2}}<0,$$

with $0<b<\alpha_{1}<\alpha_{2}$. The analysis is analogous to the proof of Proposition 3. □

After analyzing the stability of disease-free points, we focus on the endemic point, where infected tumor cells and virions coexist in equilibrium with infection-free tumor cells. This critical point is particularly interesting, as its analysis may reveal other dynamic behaviors such as periodic oscillations through a Hopf bifurcation. The endemic point in the system (31) is $P_{eH}=\left(T_{eH},I_{eH},V_{eH}\right)$, and in particular

(33) $$T_{eH}=\left(\frac{1}{b}\right)\frac{1}{R_{0}^{2}},\;I_{eH}=\left(\frac{\lambda }{b\mu }\right)\frac{\left(R_{0}^{2}-1\right)\left(R_{\alpha_{1}}^{2}-1\right)\left(R_{\alpha_{2}}^{2}-1\right)}{R_{0}^{2}R_{\alpha_{1}}^{2}R_{\alpha_{2}}^{2}},\;V_{eH}=\left(\frac{\lambda }{\beta }\right)\frac{\left(R_{0}^{2}-1\right)\left(R_{\alpha_{1}}^{2}-1\right)\left(R_{\alpha_{2}}^{2}-1\right)}{R_{0}^{2}R_{\alpha_{1}}^{2}R_{\alpha_{2}}^{2}}.$$

The existence conditions for the point are two main cases $1<R_{0}^{2}<\alpha_{1}/b$ or $R_{0}^{2}>\alpha_{2}/b$.

Following the model approach with a strong Allee effect, we will examine the Hopf bifurcations considering their dependence on the reproductive number (19) and the parameters (32) at the critical point $P_{eH}$ (33).

**Theorem 5**. *Consider the system* (31) *and the critical point* $P_{eH}$ (33). *The following set*

(34) $$H_{\text{Hyper}}=\left\{\left(b,\alpha_{1},\alpha_{2},\delta ,,\lambda ,\mu ,R_{0}^{2},\Phi_{1},\Phi_{2}\right)|\lambda \Phi_{1}-\Phi_{2}=0\right\},$$

contains the symmetric-saddles and Hopf bifurcations, with

(35) $$\Phi_{1}=(\delta +\mu ){\left(b^{2}R_{0}^{4}+b\left(R_{0}^{2}-2\right)R_{0}^{2}\left(\alpha_{1}+\alpha_{2}\right)+\alpha_{1}\alpha_{2}\left(3-2R_{0}^{2}\right)\right)}^{2}\;\Phi_{2}=b^{2}R_{0}^{4}\left(-\delta^{2}-\mu^{2}+\delta \mu \left(R_{0}^{2}-3\right)\right)-bR_{0}^{2}\left(\alpha_{1}+\alpha_{2}\right)\left(\delta^{2}\left(R_{0}^{2}-2\right)\right.\;\left.+\delta \mu \left(3R_{0}^{2}-5\right)+\mu^{2}\left(R_{0}^{2}-2\right)\right)+\alpha_{1}\alpha_{2}\left(\delta^{2}\left(2R_{0}^{2}-3\right)+\delta \mu \left(5R_{0}^{2}-7\right)\right.\;\left.+\mu^{2}\left(2R_{0}^{2}-3\right)\right)b^{2}R_{0}^{6}.$$

**Proof**. Taking the endemic point $P_{eH}$ (33), sufficient conditions are sought for the Hopf bifurcation, and the analysis is analogous to the proof of Theorem 4 to obtain the expression (34). □

Once the existence of a Hopf bifurcation is confirmed, we will analyze the stability of the endemic point and determine the conditions under which it is stable or unstable.

**Proposition 7**. *Let* $1<R_{0}^{2}<\alpha_{1}/b$ *or* $R_{0}^{2}>\alpha_{2}/b$ *be the condition of existence of the endemic critical point* $P_{eH}$ (33), *and then it is stable, where* $\lambda \Phi_{1}<\Phi_{2}^{2}$ *as in* (34).

**Proof**. By linearizing the system (31) and obtaining the Jacobian matrix A, its characteristic polynomial $P(\psi {)}_{H}$, and the coefficients $a_{1_{H}},a_{2_{H}}$, and $a_{3_{H}}$ at the point $P_{eH}$ (33) we obtain the following.


$$a_{1_{H}}=\left(3R_{0}^{2}-4\right)R_{\alpha_{1}}^{2}R_{\alpha_{2}}^{2}\alpha_{1}\alpha_{2}\lambda \;+\left(3-2R_{0}^{2}\right)bR_{0}^{2}R_{\alpha_{1}}^{2}R_{\alpha_{2}}^{2}\left(\alpha_{1}+\alpha_{2}\right)\lambda \;-\left(\left(R_{\alpha_{1}}^{2}\left(1+R_{\alpha_{2}}^{2}\right)\right)+\left(R_{\alpha_{2}}^{2}-1\right)\right)b^{2}R_{0}^{4}\lambda \;-\left(R_{\alpha_{1}}^{2}\left(R_{\alpha_{2}}^{2}(\delta +\mu )-\lambda \right)-\left(R_{\alpha_{2}}^{2}-1\right)\lambda \right)R_{0}^{6}b^{2}.\;a_{2_{H}}=\left(\left(R_{0}^{2}-1\right)\left(1-R_{\alpha_{1}}^{2}\right)+\left(\left(1-R_{0}^{2}\right)+R_{\alpha_{1}^{2}}\right)R_{\alpha_{2}^{2}}\right)(\delta +\mu )\lambda \;+\left(4-3R_{0}^{2}\right)R_{\alpha_{1}}^{2}R_{\alpha_{2}}\alpha_{1}\alpha_{2}(\delta +\mu )\lambda \;+\left(2R_{0}^{2}-3\right)R_{0}^{2}R_{\alpha_{1}}^{2}R_{\alpha_{2}}b\left(\alpha_{1}+\alpha_{2}\right)(\delta +\mu )\lambda \;a_{3_{H}}=\left(R_{0}^{2}-1\right)\left(R_{\alpha_{1}}^{2}-1\right)\left(R_{\alpha_{2}}^{2}-1\right)R_{0}^{6}b^{3}\delta \mu .$$

To prove the stability of $P_{eH}$ (33), we need to check that $\left.\left(a_{1_{H}}a_{2_{H}}-a_{3_{H}}\right)\right)>0$. If we use (34), let ϵ such that

(36) $$\lambda =\epsilon \frac{\Phi_{2}^{2}}{\Phi_{1}},$$

and we obtain

$$\left(a_{1_{H}}a_{2_{H}}-a_{3_{H}}\right)=\epsilon \left(\epsilon -1\right)\frac{\Phi_{2}^{2}}{\Phi_{1}}.$$

If ϵ=0, then λ=0 is not a positive range value. If ϵ=1, then we recover the set (34) and we have $\left(a_{1_{H}}a_{2_{H}}-a_{3_{H}}\right)=0$. In the case of ϵ>1 we have $\left(a_{1_{H}}a_{2_{H}}-a_{3_{H}}\right)>0$, so $P_{eH}$ (33) is stable when

$$\lambda \Phi_{1}=\Phi_{2}^{2}<\epsilon \Phi_{2}^{2},$$

for $1<R_{0}^{2}<\alpha_{1}/b$ or $R_{0}^{2}>\alpha_{2}/b$, and $a_{1H},a_{2H},a_{3H}>0$. □

**Figure 7.**
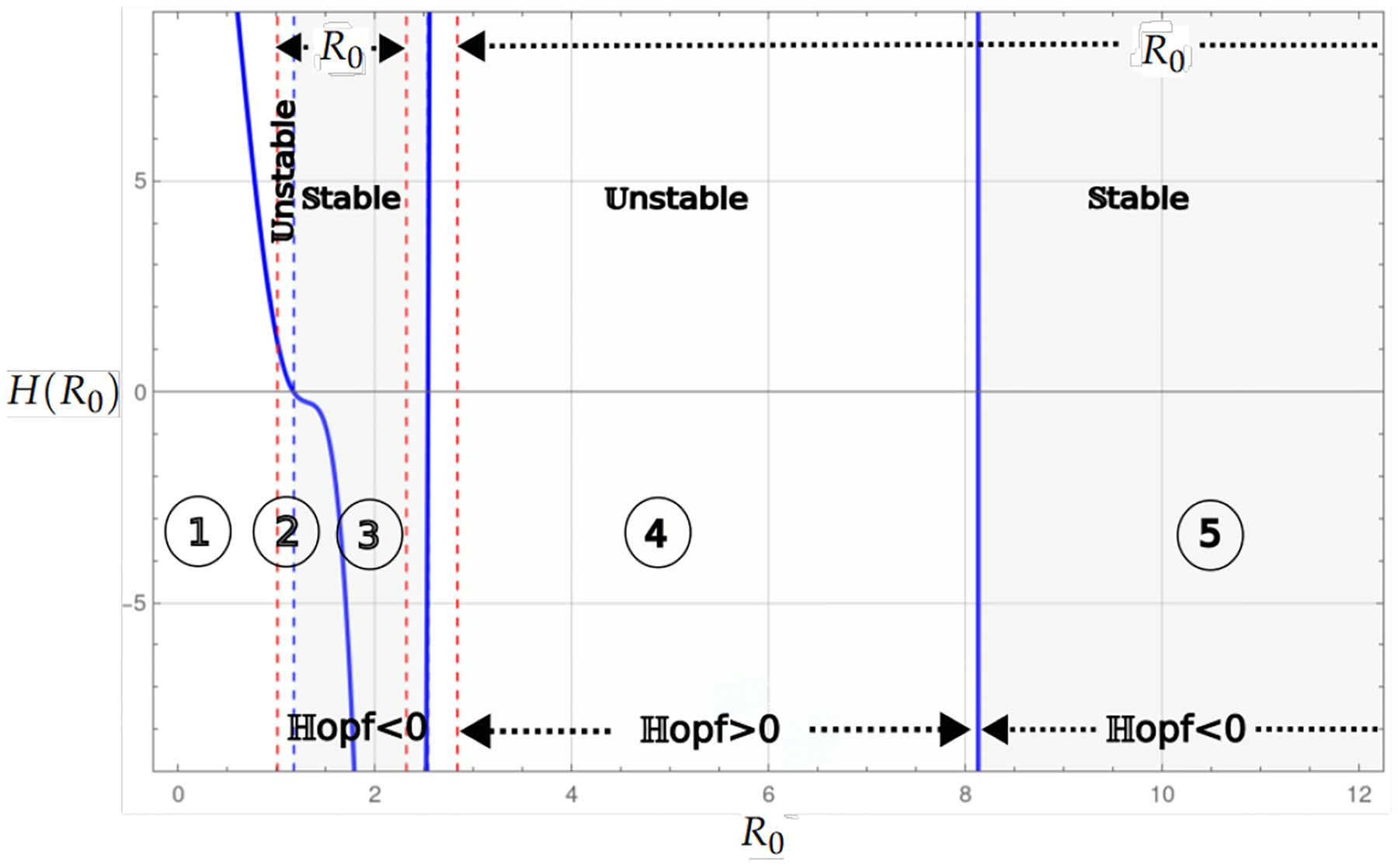
The bifurcation diagram corresponds to the model with logistic growth and the hyper Allee effect (31). The blue curve represents a projection of the Hopf hyperparametric set (34) as a function of $R_{0}$.

For the Allee hyper effect, we have two threshold values, which satisfy $b<\alpha_{1}<\alpha_{2}$; there are levels $R_{\alpha_{2}}<R_{\alpha_{1}}<R_{0}$ that largely determine the dynamics of the system with logistic growth. Theorem 5 and Proposition 7 determine the stability of the system, as seen in the bifurcation diagram in Figure 7, for the conditions of existence of the endemic critical point $1<R_{0}<\alpha_{1}/b$ or $R_{0}^{2}>\alpha_{2}/b$, indicated by the vertical red dashed lines. In both intervals, one can distinguish between stability and instability through the Hopf bifurcation curve in blue. There are cycles in a neighborhood where the Hopf curve is zero, which determines latency in cancer. In addition, the population can be highly endemic with a high level of $R_{0}$, as seen in Region 5 of the bifurcation diagram.

## 5. Bifurcation Diagrams and Numerical Results

Using bifurcation diagrams and numerical continuation techniques complements the qualitative analysis of system dynamics. These methods allow us to visualize how the variation of critical parameters, in this case, the basic reproductive numbers, affects the existence and stability of equilibrium points and periodic solutions of the model. The bifurcation diagrams offer a graphical representation of dynamics in the system, such as Hopf bifurcations, which enable us to find some changes in the stability of critical points, providing a global perspective on the behavior of the system. In turn, numerical continuation allows us to trace these solutions over multiple parametric values, facilitating the identification of regions of stability, instability, or complex oscillations.

### 5.1. Bifurcation Diagrams

In the following, we present the diagrams obtained from the analytical results of the previous two sections.

Figure 2 shows the bifurcation diagram corresponding to the system, which incorporates linear growth with a strong Allee effect. The bifurcation parameter considered is $R_{\alpha }$ from (3). In the upper part of the diagram, the existence interval of the critical endemic point, defined by $0<R_{\alpha }<1$, is shown, delimited by two dashed red vertical lines. The curve in blue corresponds to the Hopf bifurcation, marking a transition of stability.

The regions identified in the diagram are as follows; Region 1 corresponds to the interval where the endemic point exists but is unstable, and Region 2 represents the interval where the endemic point exists and is stable. Region 3 is associated with the region where the Hopf curve takes negative values; however, the values associated with the endemic critical point are not biologically admissible, while in the last region in the Figure 2, the Hopf curve takes positive values and has no biological interpretation in the model’s context.

Figure 3 shows the bifurcation diagram for the case where the model simultaneously considers linear growth with the strong and weak Allee effects. These regions of stability are separated by the blue dashed vertical line, which marks the transition of the Hopf curve from positive to negative values.

Figure 4 shows the bifurcation diagram for the system with linear growth and hyperbolic Allee effects. A prominent feature of this model is the presence of two regions of existence for the endemic point, which are delimited by red-dashed vertical lines. The bluedashed vertical lines represent the transition of the Hopf curve from positive to negative values. When the Hopf curve is below the horizontal axis, there may be stability at the endemic point. The regions identified in the diagram are as follows: In Region 1, the existence of the endemic point is seen as the Hopf curve is negative, and the endemic point is stable. In Region 2, the Hopf is positive, and then the endemic point is unstable. The endemic point does not exist in Regions 3 and 4. Regions 5 and 6 are stable and unstable.

Figure 5 shows the bifurcation diagram for the logistic growth system without the Allee effect. Region 1 corresponds to the case where the endemic point is absent. Region 2 is the stable region where the endemic point exists, and the values of the Hopf curve are negative. Finally, Region 3 shows the values for $R_{0}$ where the endemic point is unstable.

**Figure 8.**
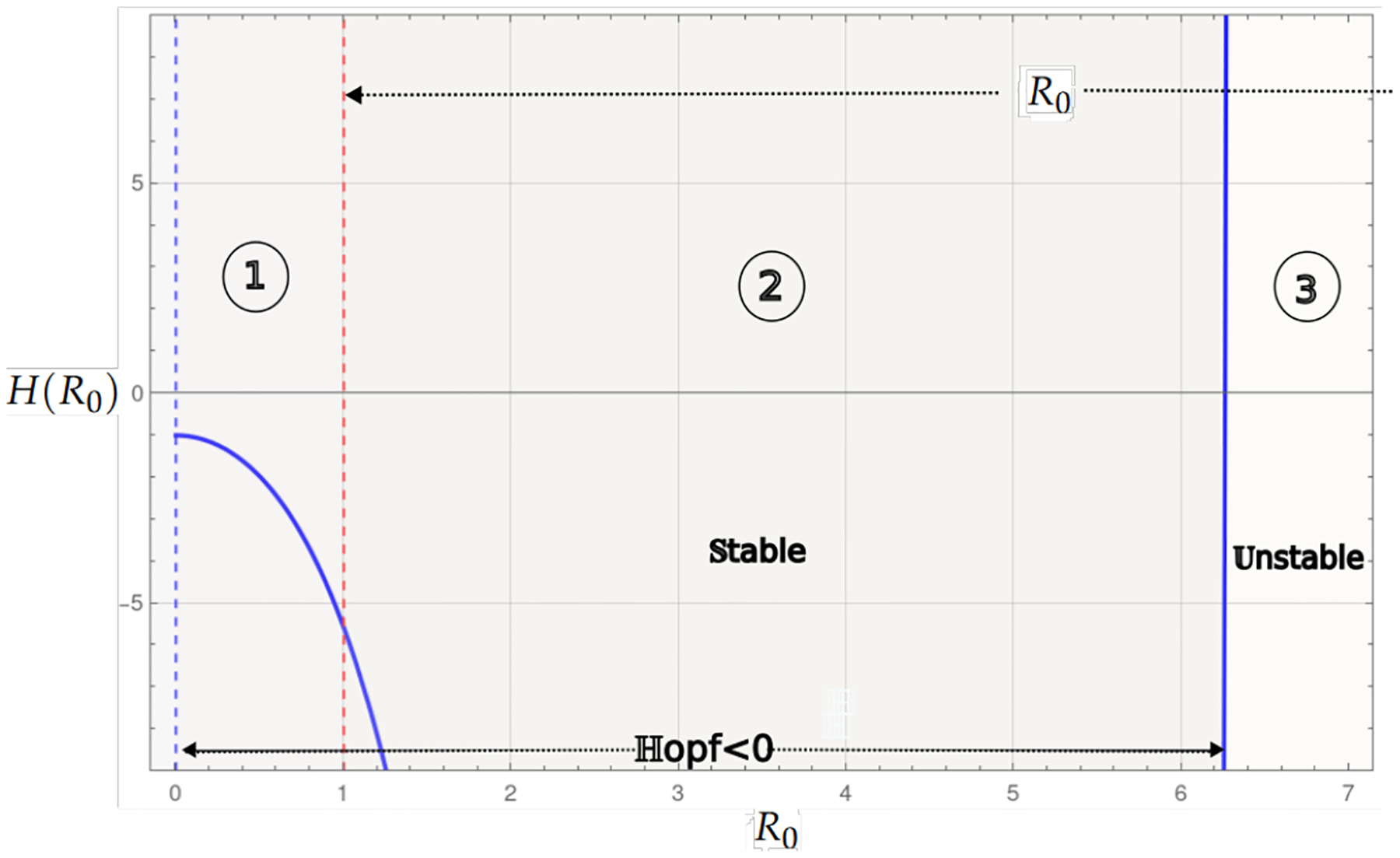
The bifurcation diagram corresponds to the model with logistic growth and weak Allee effect. The blue curve represents a projection of the Hopf hyperparametric set as a function of $R_{0}$.

Figure 8, similar to the previous case, shows the bifurcation diagram for the system with logistic growth and a weak Allee effect. Region 1 corresponds to the case in which the endemic point is not present. Region 2 is the stable region, where the endemic point exists and the values of the Hopf curve are negative. Finally, Region 3 shows the values for $R_{0}$, where the endemic point is unstable. The Allee effect benefits the stable equilibrium point.

**Figure 9.**
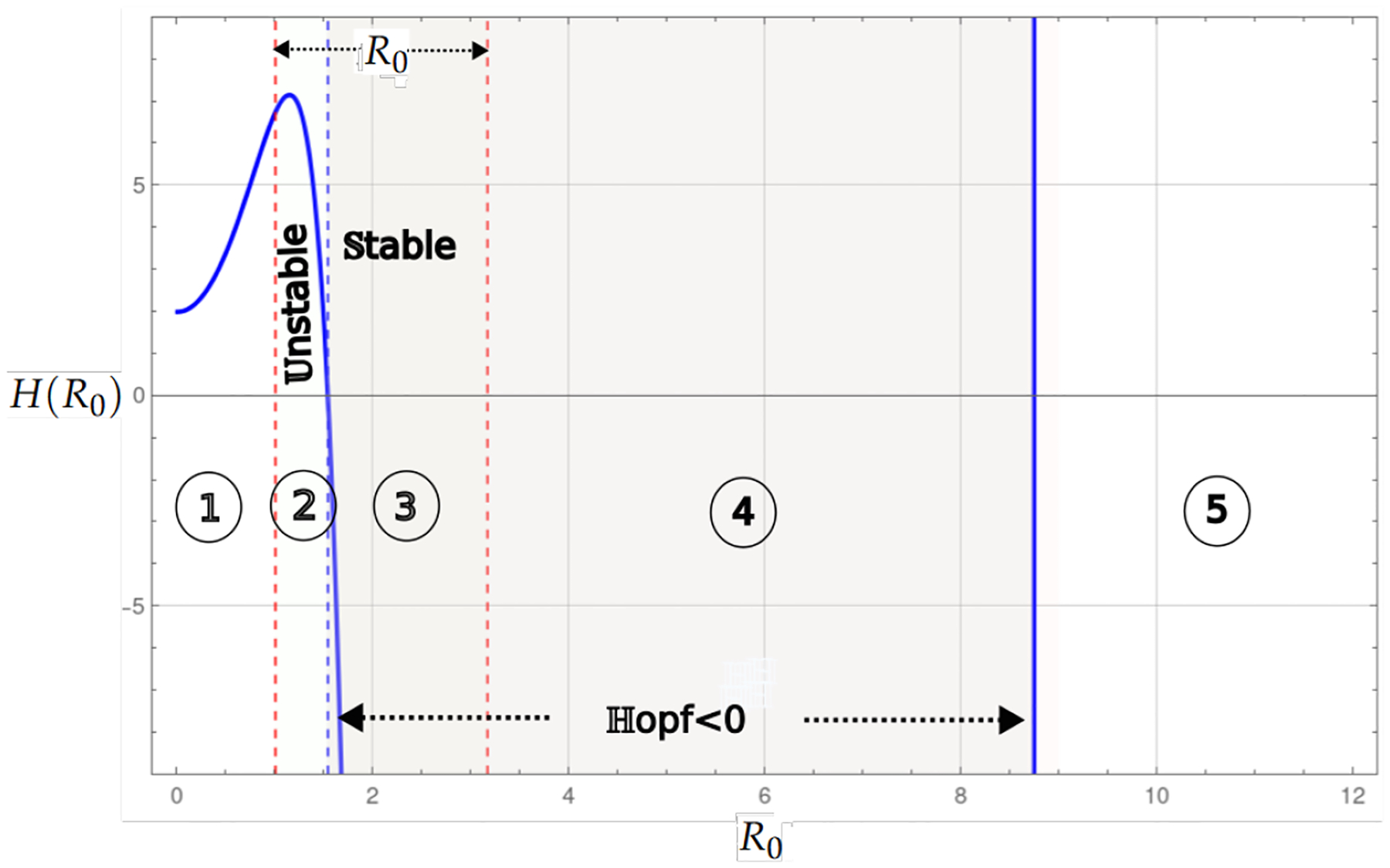
The bifurcation diagram corresponds to the model with logistic growth and both weak and strong Allee effects. The blue curve represents a projection of the Hopf hyperparametric set as a function of $R_{0}$.

Figure 9 shows the bifurcation diagram for the system with logistic growth and simultaneous weak and strong Allee effects. Region 1 corresponds to the case of the nonexistence of the endemic point. Region 2 is the region where the endemic point is unstable. Region 3 shows the values for $R_{0}$, where the endemic point is stable. Region 4 is the region where the endemic point does not exist, although the Hopf curve has negative values. In Region 5, there is also no biological interpretation.

Figure 6, similar to the previous case, shows the bifurcation diagram for the system with logistic growth, but only with the strong Allee effect. Region 1 corresponds to the case of the nonexistence of the endemic point. Region 2 is the region where the endemic point exists and is unstable, and Region 3 shows the values for $R_{0}$, where the endemic point exists and is stable. Region 4 is the region where the endemic point does not exist, although the Hopf curve has negative values, and in Region 5, there is no biological interpretation. Figure 7 shows the bifurcation diagram for the model with logistic growth and the Allee hyper effect. There is no endemic point in Region 1 and in the region between 3 and 4. In Regions 2 and 4, the endemic point is unstable, while in Regions 3 and 5, it is stable.

### 5.2. Numerical Continuation

**Figure 10.**
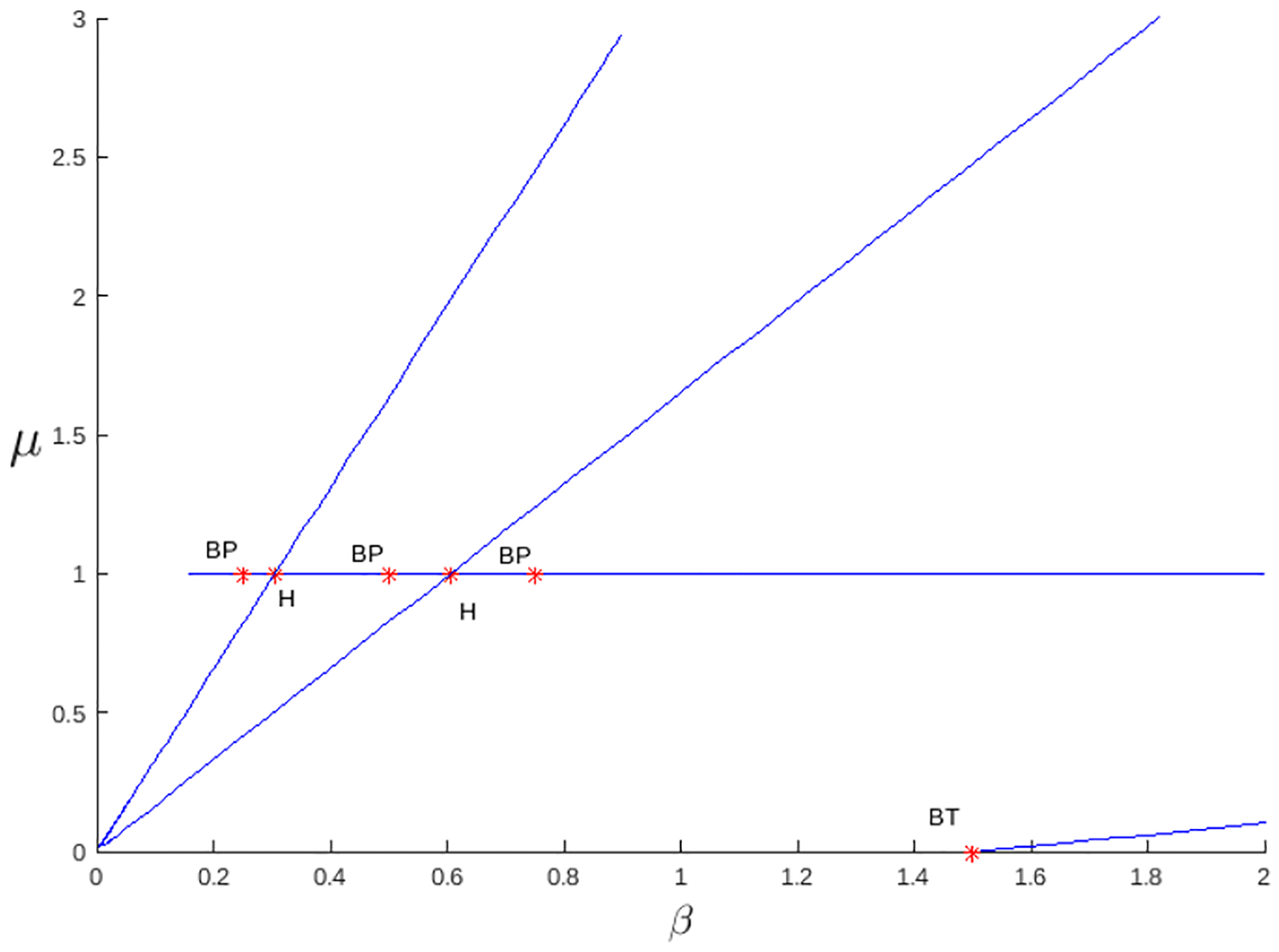
Numerical continuation in MATLAB (24.1.0.2653294)-MATCONT (7.1) of the bifurcation diagram for the model with logistic growth and hyper Allee effect. The blue horizontal line is a continuation through critical endemic points. The *BPs* (Branch Points) mark changes in the solution structure, determining the existence by sign changes in some components of the endemic equilibrium (33). The H points represent Hopf bifurcations (34), indicated by the diagonal lines as a function of the parameters (β,μ). Finally, *BT* corresponds to a Bogdanov–Takens bifurcation, with no oncological interpretation due to its negative μ component.

**Figure 11.**
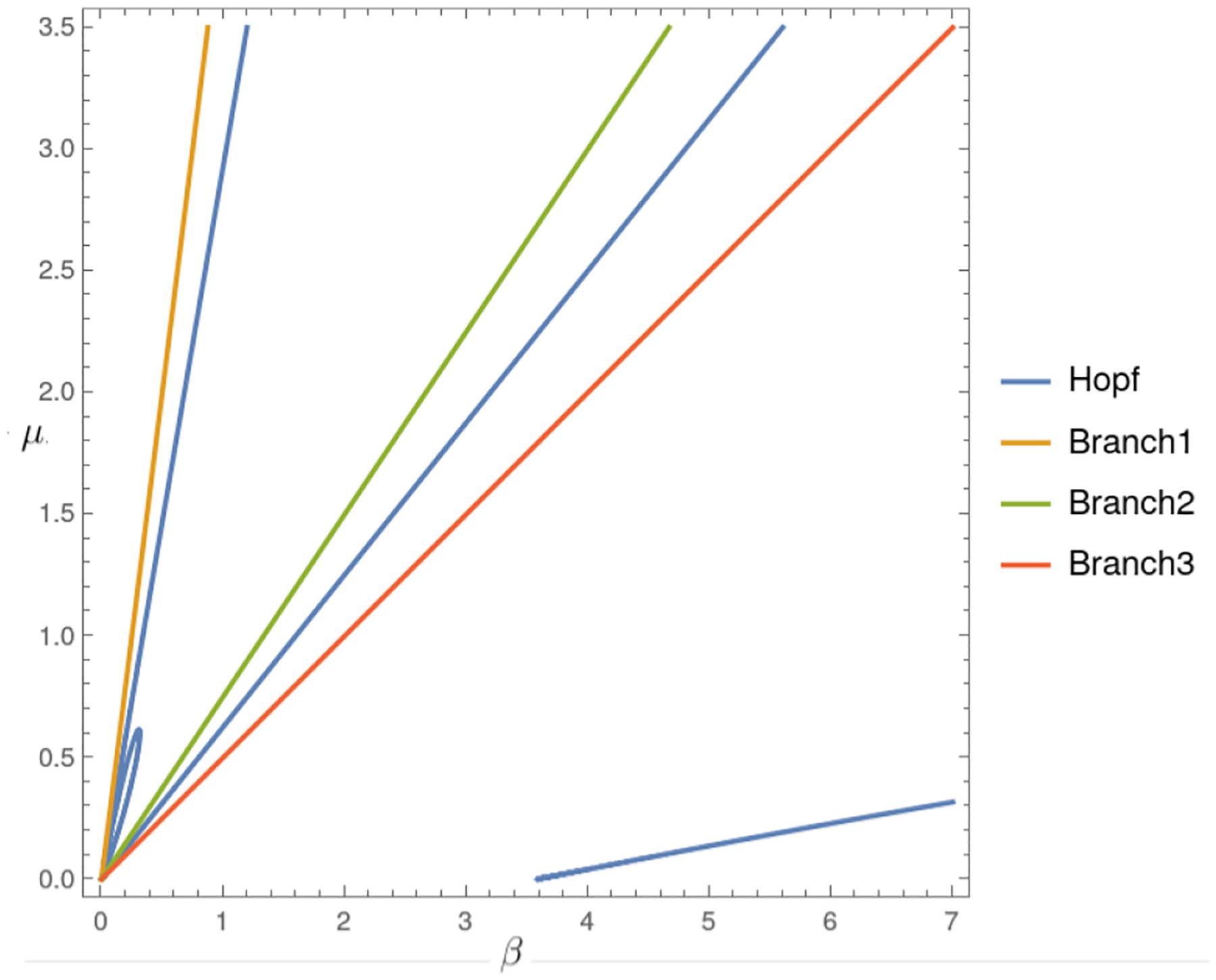
The projection of the analytical sets in the bifurcation diagram corresponds to the model with logistic growth and the hyper Allee effect.

Figure 10 shows the numerical continuation, performed in MATLAB (24.1.0.2653294)-MATCONT (7.1) [47], of the critical points in terms of the parameters β and μ. The initial values used for numerical continuation are $\lambda =1,b=1/4,\alpha_{1}=1/2,\alpha_{2}=3/4,p=1,\delta =1,\beta =0.3,\mu =1$. On the other hand, Figure 11 presents the projection of the Hopf curve, defined in (34), in terms of the exact parameters β and μ. The latter plot is equivalent to the bifurcation diagram presented in Figure 7, with clear correspondences between the highlighted elements; Branch 1, marked in orange in Figure 11, corresponds to the first dashed vertical line in Figure 7 and is in condition $R_{0}^{2}=1$. Branch 2, represented by the green straight line in Figure 11, is associated with the second dashed vertical line in Figure 7 (from left to right), corresponding to condition $R_{0}<\alpha_{1}/b$. The red line in Figure 11 represents the third red dashed vertical line in Figure 7, defined by condition $R_{0}^{2}>\alpha_{2}/b$. The Hopf projection in blue in Figure 11 is given by Equation (34) and describes the stability transitions through the parameters β and μ. In this context, Figure 10 provides a numerical representation equivalent to Figure 11, which consistently supports the analytical results obtained previously.

**Figure 12.**
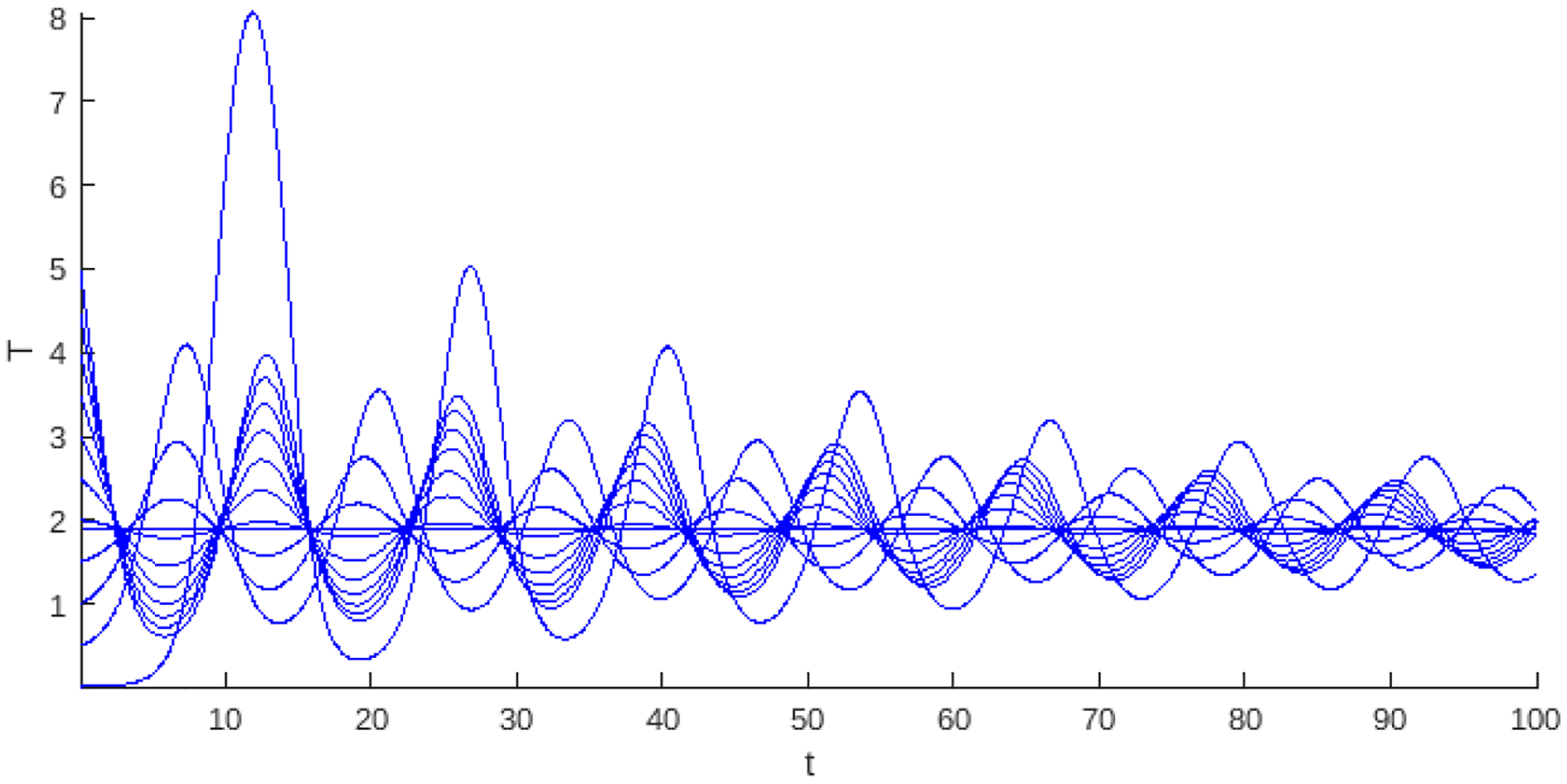
The numerical solution of a stable endemic point corresponds to the model with logistic growth and no Allee effect, where the different curves represent different initial conditions. Parameter values: λ=1,b=1/10,α=0,ω=0,β=0.2657,μ=0.5,p=1, and δ=1.

**Figure 13.**
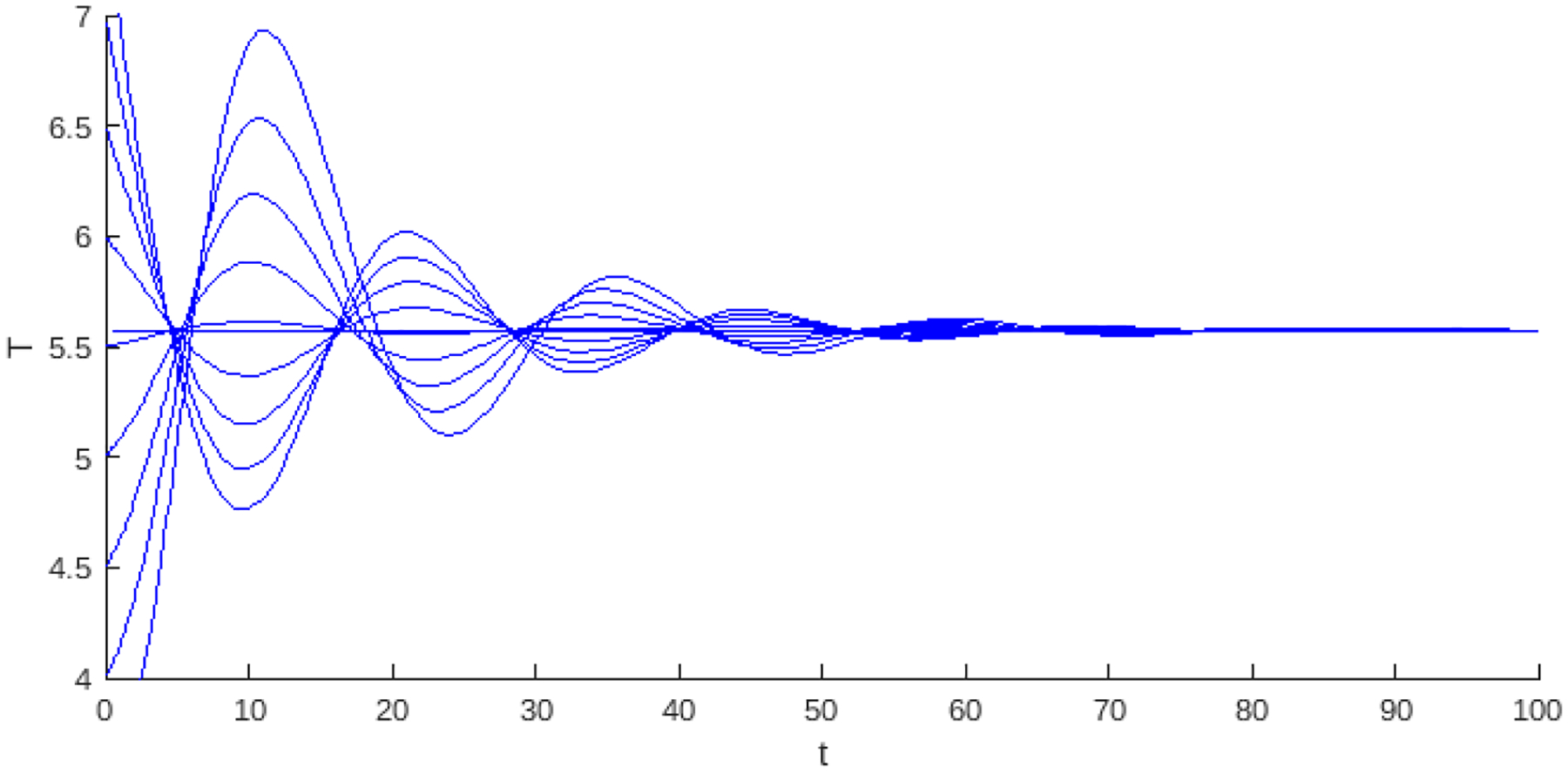
Numerical solution of a stable endemic point corresponds to the model with logistic growth and with weak Allee effect, with different curves corresponding to different initial conditions. Parameter values: λ=1,b=1/10,α=0,ω=5,β=0.0897,μ=0.5,p=1, and δ=1.

**Figure 14.**
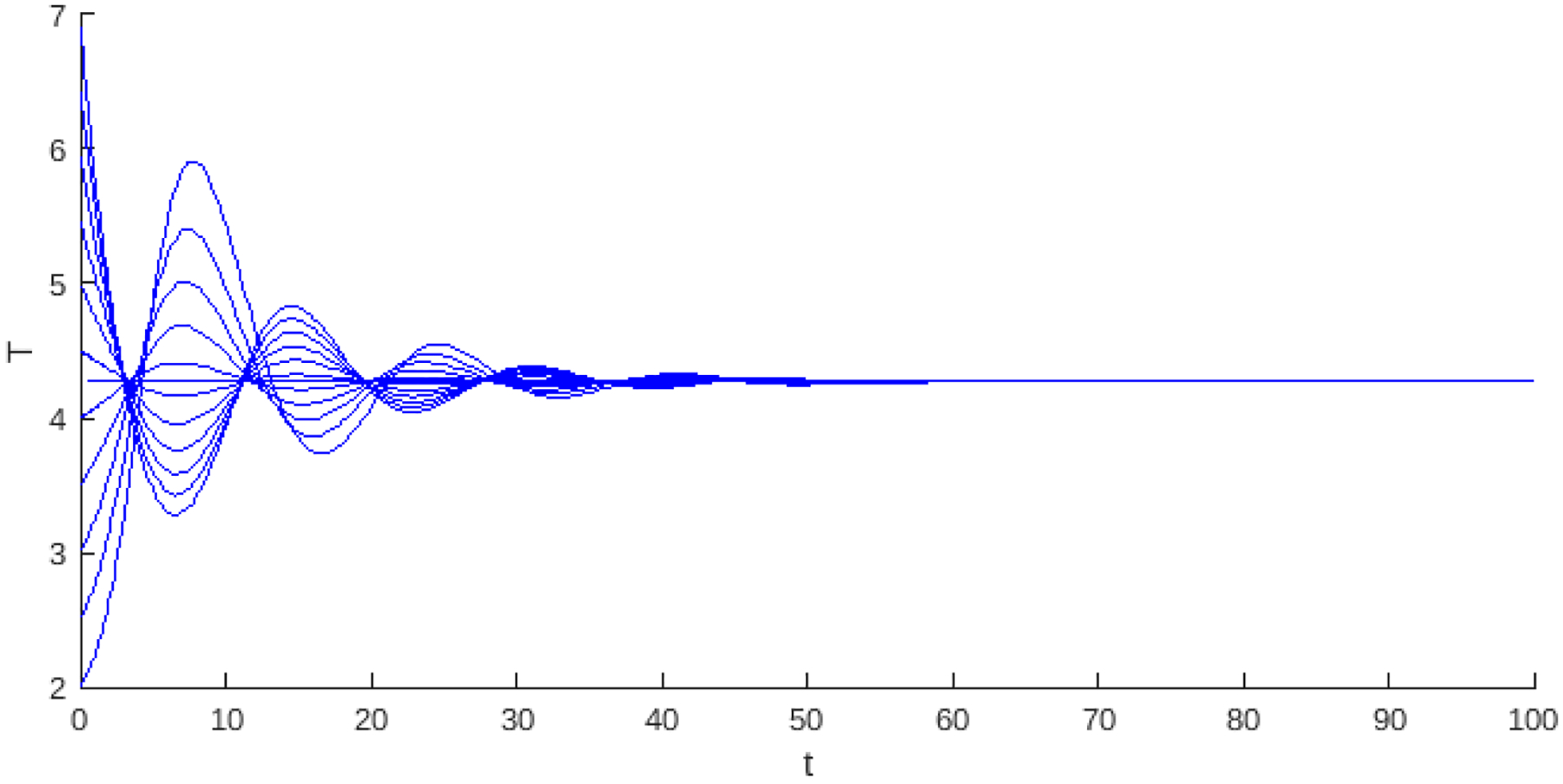
The numerical solution of a stable endemic point corresponds to model (24) with logistic growth and a strong Allee effect, where different curves correspond to different initial conditions. Parameter values: λ=1,b=1/10,α=0.5,ω=0,β=0.117,μ=0.5,p=1, and δ=1.

**Figure 15.**
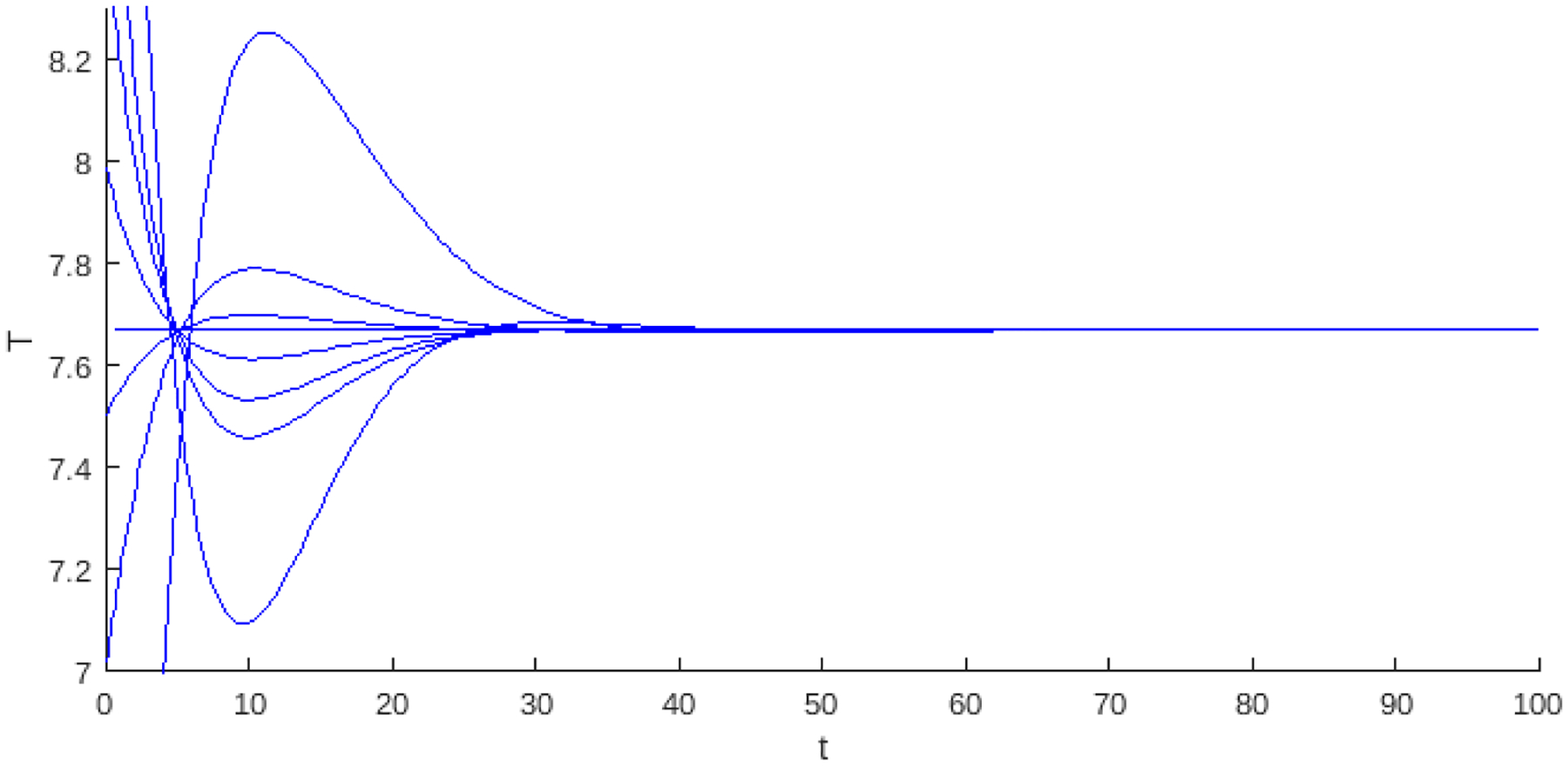
The numerical solution of a stable endemic point corresponds to the model with logistic growth, with both weak and strong Allee effects, where different curves correspond to different initial conditions. Parameter values λ=1,b=1/10,α=0.5,ω=5,β=0.0652,μ=0.5,p=1, and δ=1.

**Figure 16.**
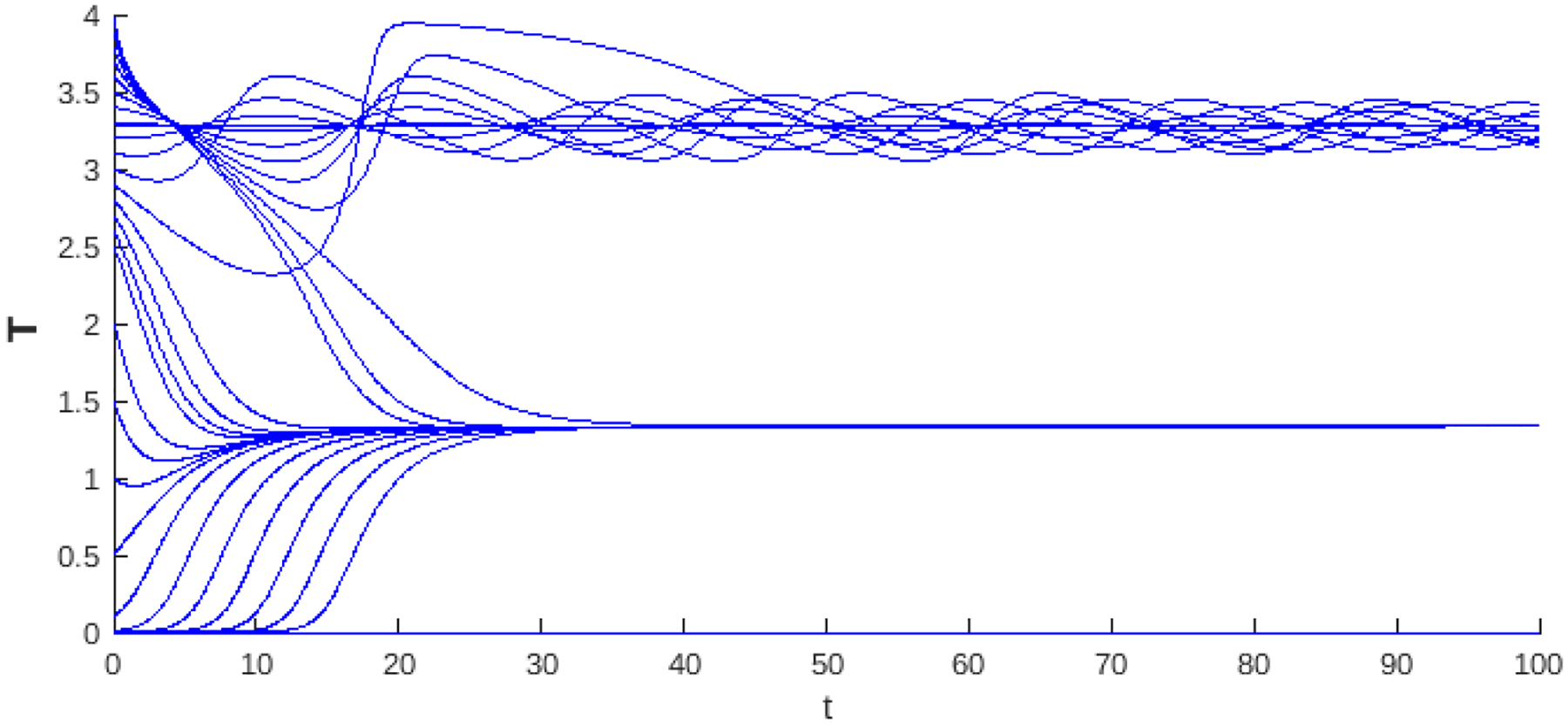
The numerical solution of endemic stability corresponds to the model (31) with logistic growth and the hyper Allee effect, where different curves correspond to different initial conditions. Parameter values: $\lambda =1,b=1/4,\alpha_{1}=1/2,\alpha_{2}=3/4,\beta =0.30551595923505,\mu =1,p=1$, and δ=1.

Figures 12–15 present the numerical solution of a stable endemic point for different values of weak and strong Allee effects in the logistic growth model. These points were selected using the parameters corresponding to stability in the bifurcation diagram shown in Figures 5–7. The numerical continuation confirms the stability of the endemic point under the specific conditions of this region, supporting the analytical results and the bifurcation diagrams previously discussed. This representation highlights how the parametric configurations within this region allow steady-state to be achieved in the dynamical system. On the other hand, Figure 16 shows a case of stability under the Allee hyper effect. Each of these levels of the cancer cell population persists without being completely extinguished. The figure shows that the initial conditions determine the long-term population level. In this case, to reach the high population level, the initial conditions must be in a small neighborhood of the highest stable population level, while for the low population level, the initial conditions can take a larger set of values. The figure shows that an initial condition close to the carrying capacity is governed by the lowest stable level, not the highest level, as expected. Under these parameter values, the basic reproductive number is $R_{0}=1.10547$, corresponding to Region 3 in the bifurcation diagram 7.

## 6. Discussion

In the base model (1) with linear growth, we note that the system cannot adequately capture the stability of the critical points by not considering more complex or realistic situations, such as Allee effects. In particular, without integrating the Allee effects or considering only a weak Allee effect, the critical point of the endemic becomes unstable. However, with a strong Allee effect, the dynamic changes. This term generates a change in the stability of the endemic critical point, accompanied by a Hopf bifurcation. This bifurcation gives rise to limit cycles, adding complexity to the system dynamics. This pattern of stability change and bifurcation is preserved even when considering strong and weak Allee effects simultaneously (2), which underlines the robustness of these dynamics in the context of the model. When we incorporate the hyperbolic Allee effect, new characteristics are introduced to the system. This type of effect defines two critical levels, $\alpha_{1}$ and $\alpha_{2}$, which determine the existence of the endemic critical point and amplify the range of stability when $\alpha_{1}<\alpha_{2}$. In this particular case (10), in the limit of $\alpha_{2}\to \alpha_{1}$, the model once again reduces to the strong Allee effect, confirming the consistency of the mathematical framework.

The logistic growth model provides a more realistic situation of tumor dynamics by incorporating the carrying capacity of the system, a factor important in biological contexts where resources are limited. From the outset, this model presents richer dynamics compared to the linear case, since even without Allee effects, a change in stability is observed through a Hopf bifurcation (Figure 5). When a weak Allee effect is incorporated, the Hopf bifurcation that gives rise to the stability change of the endemic critical point is preserved. On the other hand, the stability region for this point becomes larger, as can be seen in Figure 8. Then, the weak Allee effect provides stability with many conditions without significantly altering the transitions of dynamics in the system. In the presence of a strong Allee effect, or when strong and weak Allee effects are considered simultaneously, the existence of the endemic critical point is reduced. This may be attributed to the imposition of a survival threshold for tumor cells, which limits their viability at low densities. Despite this reduction in the existence of the endemic point, the stability change is maintained by a Hopf bifurcation (Figures 6 and 9). The case with the hyperbolic Allee effect (Figure 6) introduces a markedly different dynamic. Here, a wider range is observed for both the existence of the endemic critical point and its stability.

The analysis results highlight the key role of the carrying capacity and the Allee effect in the dynamics of the system, particularly in stabilizing the critical endemic point. The carrying capacity defines a range of stability in the system for values of the basic reproduction number $1<R_{0}^{2}<\chi$, where χ is determined by the threshold associated with the Hopf bifurcation. This range indicates that, under controlled conditions, the system can maintain a stable equilibrium without complete tumor eradication. The Allee effect, on the other hand, reduces the existence of a critical endemic point for $1<R_{0}^{2}<1/(b\alpha )$ levels, promoting the propagation of the virus and consequently reducing the population of cancer cells. This phenomenon suggests that the Allee effect may act as a regulatory mechanism that favors tumor control through viral propagation. In the case of the hyper Allee effect, the endemic point persists in an extended range for $1<R_{0}^{2}<\alpha_{1}/b$, further facilitating the virus’s spread. When $R_{0}^{2}>\alpha_{2}/b$, as observed in Region 5 (Figure 7), the dynamics indicate a possible drastic reduction in the tumor population. This region could represent a favorable scenario for antitumor therapies, as it combines effective virus propagation with a significant impact on cancer cell reduction.

The weak Allee effect explored in this study is observed to facilitate the stability of the endemic critical point, expanding the stability region without significantly altering the dynamic transitions of the system, meaning that a low density of virus-infected tumor cells could still allow cancer proliferation, but with lower efficiency. However, the strong Allee effect with logistic growth indicates that if the number of infected tumor cells is insufficient, the viral infection does not spread effectively, leading to tumor eradication. However, if the cell density exceeds the threshold, the tumor may persist or grow, as shown in Figure 6. When the weak and strong Allee effects are combined, the existence of the endemic critical point can be limited. In fact, in these observed cases, the limitation is very marked depending on the basic reproductive number (see Figures 3 and 9).

In Allee, the hyper effect is a more extreme case, where there are two critical thresholds, $\alpha_{1}$ and $\alpha_{2}$, a lower one, below which the tumor population collapses, and an upper one, which defines a steady state in which the tumor can persist. This effect suggests that the virus could be more effective in eliminating cancer if the cell population is kept within certain limits, $R_{0}^{2}>\alpha_{2}/b$. In this region, where the number of infected cells is high, there is a reduction in the tumor population, which could represent an advantage for oncolytic therapies.

This study is theoretical and hypothetical, as its primary objective was to explore the dynamics through bifurcations rather than fitting specific experimental data at this stage. However, experimental studies in cancer have empirically verified its behavior [14,16,48–53], supporting the relevance of incorporating different types of Allee effects into mathematical models, such as the one presented here, in the context of population dynamics under oncolytic virotherapy. Other papers support the idea, with experimental data showing that cancer can be modeled, including Allee effects. For example, in [48], in his discussion section paragraph 6, they include the case of the hyper Allee effect. At the same time, the works [12,13,15,54] show that populations at low densities cannot proliferate as easily, according to an Allee effect in cancer. Other references supporting the use of Allee effects in the epidemiology model are listed in Table 1. This study can be extended in future research by calibrating the model with experimental data.

The weak Allee effect typically describes the situation in which the population is small and still able to grow. In the base model (1) we addressed, this effect allows the virus to infect and control tumor growth without necessarily eradicating it completely. The strong Allee effect needs to overcome a population level to avoid extinction, which in this context means that the tumor cell population is sufficiently high to avoid extinction. The combined effects mean that the tumor can reach a dormant state with viruses or be eliminated if the population is small enough, so the relatively high stable critical point in Figure 15 is observed. The most complex case of the Allee effect is hyper since two parameters are needed to determine the complex dynamics of the system. In the case of instability, if the population is below the first parameter $1/\alpha_{2}$, then the tumor would be eradicated; if the population is above the second threshold $1/\alpha_{1}$, it can proliferate and the tumor grows uncontrollably, but in the case of stability, for the basic reproductive number $R_{0}$ with values that are associated with negative Hopf curves such as Regions 3 or 5 in Figure 7, the Allee hyper-threshold values would cause endemic bistability as in Figure 16.

## 7. Conclusions

Our exploration of modeling in oncological virotherapy includes an epidemiology model with linear and logistic growth, with different Allee effects to observe their behavior in the dynamics of interaction with viruses. There are different modeling approaches, such as models with fractional operators, as is the case of [26], which, without considering the Allee effect, obtains complex dynamics but presents problems in analyzing stability. On the other hand, [4] discusses different modeling approaches in virology, including spatio-temporal models with PDE, epidemiology models, and variants of these. However, they do not include any Allee effect. In works such as [29–31], the authors thoroughly study partial derivative modeling for oncological virotherapy modeling, where they couple tumor growth with cell–virus–immunity interaction; they also do not include the Allee effect. In [32], they consider an epidemiological model with the Allee effect, but have not yet done this in cancer. On the other hand, works such as [28,57–59], are conceptually closer to our approach to analyzing dynamics and stability, but also do not include the Allee effect.

Through theoretical analysis and bifurcation diagrams, critical conditions that determine the existence and stability of equilibrium points and periodic oscillations through Hopf bifurcations have been identified. The results highlight that first, the inclusion of the Allee effect, whether weak, strong, or hyper, significantly alters the dynamics of the system. In particular, strong and hyper effects introduce critical population thresholds that influence tumor eradication or persistence. The bifurcation diagrams we provide in this work are tools for identifying regions of stability and instability and help us understand dynamic transitions between disease-free and endemic states. In addition, when we incorporate logistic growth, we obtain differences in dynamics versus the linear growth model. In addition, we can observe that the different Allee effects affect the stability of the base model, too. All these combinations of factors should allow us to design viral cancer therapies with strategies based on critical parameters such as reproduction rates, Allee effects, and carrying capacity.

Our main contribution lies in the inclusion of different Allee effects using an epidemiological modeling approach for tumor–virus–immunity interactions, where we have analyzed their impact on the conditions of the existence of endemic critical points as well as their stability and bifurcation properties. Future research could extend this analysis to more complex models, including spatial heterogeneity, variability in the immune response, or treatment combination, and allow us to validate results with real data and fit the parameters of the system.

## Figures and Tables

**Table 1.** Results for different models, growth types, Allee effects, ranges of $R_{0}$, and relevant references for Allee effects.

Model+Growth	Allee Effect	Range $R_{0}$	Hopf	References
Model 1 Linear	None	N/A	No	[15,55]
Model 2 Linear	Weak	N/A	No	[15,55]
Model 3 Linear	Strong	$0<R_{\alpha }^{2}<1$	Yes	[13,15,55]
Model 4 Linear (2)	Weak + Strong	$0<R_{\alpha }^{2}<1$	Yes (5)	[13,15,55]
Model 5 Linear (10)	Hyper	$0<R_{0}^{2}<1/\alpha_{2}$ or $R_{0}^{2}>1/\alpha_{1}$	Yes (13)	[12,13,15,48,54]
Model 6 Logistic (17)	None	$R_{0}^{2}>1$	Yes (22)	[15]
Model 7 Logistic	Weak	$R_{0}^{2}>1$	Yes	[12,14,15,50,56]
Model 8 Logistic (24)	Strong	$R_{\alpha }^{2}<1<R_{0}^{2}$	Yes (28)	[12,14,15,50,56]
Model 9 Logistic	Weak+Strong	$1<R_{0}^{2}<1/(b\alpha )$	Yes	[12,14,15,50,56]
Model 10 Logistic (31)	Hyper	$1<R_{0}^{2}<\alpha_{1}/b$ or $R_{0}^{2}>\alpha_{2}/b$	Yes (34)	[12,13,15,48,54]

## Data Availability

The original contributions presented in this study are included in the article. Further inquiries can be directed to the corresponding author(s).
